# A social network model of COVID-19

**DOI:** 10.1371/journal.pone.0240878

**Published:** 2020-10-29

**Authors:** Alexander Karaivanov

**Affiliations:** Department of Economics, Simon Fraser University, Burnaby, BC, Canada; Cinvestav-Merida, MEXICO

## Abstract

I construct a dynamic social-network model of the COVID-19 epidemic which embeds the SIR epidemiological model onto a graph of person-to-person interactions. The standard SIR framework assumes uniform mixing of infectious persons in the population. This abstracts from important elements of realism and locality: (i) people are more likely to interact with members of their social networks and (ii) health and economic policies can affect differentially the rate of viral transmission via a person’s social network vs. the population as a whole. The proposed network-augmented (NSIR) model allows the evaluation, via simulations, of (i) health and economic policies and outcomes for all or subset of the population: lockdown/distancing, herd immunity, testing, contact tracing; (ii) behavioral responses and/or imposing or lifting policies at specific times or conditional on observed states. I find that viral transmission over a network-connected population can proceed slower and reach lower peak than transmission via uniform mixing. Network connections introduce uncertainty and path dependence in the epidemic dynamics, with a significant role for bridge links and superspreaders. Testing and contact tracing are more effective in the network model. If lifted early, distancing policies mostly shift the infection peak into the future, with associated economic costs. Delayed or intermittent interventions or endogenous behavioral responses generate a multi-peaked infection curve, a form of ‘curve flattening’, but may have costlier economic consequences by prolonging the epidemic duration.

## 1 Introduction

I construct and compute a dynamic social network-based model of the COVID-19 epidemic and use it to evaluate a range of simulated health and economic policies—herd immunity, distancing, lockdown, testing, quarantine, and contact tracing. Endogenous behavioral responses are also analyzed. The analytical framework superimposes the classic SIR model of infectious diseases ([1–4] among many others) onto a social-network graph of interactions. Previous work on infection spread via networks is mostly theoretical and includes [5–10]. An economic module can be overlaid onto the disease dynamics, similar to [11] or [3]. Additionally, since the network model tracks individual nodes over time, heterogeneity (e.g., in savings, employment status; ability to pay rent or bills) can be incorporated.

SIR (or SEIR) Markov models characterize the spread of an epidemic over time in a population of agents who pass through the states of ‘Susceptible’, (‘Exposed’), ‘Infectious’ and ‘Resolved’ (recovered or dead). Biological and socioeconomic parameters determine the duration and transition probabilities between the states. Health and economic policies such as physical distancing, testing and quarantine also influence the spread of the disease by affecting the rate and number of contacts between agents.

The standard SIR framework assumes random (uniform) mixing of infectious persons with the rest of the population. While helpful for simplifying the dynamics and computing outcomes, this population-level random matching assumption abstracts from important elements of realism and locality: (i) people are more likely to interact with members of their social network, broadly defined (e.g., family, work, or distance based); (ii) health and economic policies targeting disease mitigation, as well as individual behavioral responses, can affect the rate of viral transmission via a person’s network of contacts vs. the population as a whole differently. For example, [12] use Facebook data and show that areas with stronger social ties to two early COVID-19 “hotspots” in the U.S. and Italy had more confirmed COVID-19 cases; (iii) social contact heterogeneity can induce path-dependence and role for ‘superspreaders’ or ‘clusters’ in the epidemic dynamics, see [13, 14].

Incorporating local, social-network based transmission in the SIR epidemiological model can yield quantitatively different outcomes and policy implications compared to the standard framework with uniform mixing. Using simulations I show that, for the same biological parameters, the standard SIR model can overstate the reproduction rate and infection peak of the epidemic. Relative to the SIR model, the network structure and degree heterogeneity introduces uncertainty and unpredictability in the epidemic dynamics and duration as well as in policy outcomes, since the infection can spread in a non-uniform, state-dependent way. The observed broad range of COVID-19 infection rates across countries, the presence of clusters and superspreaders and the prolonged plateau of new daily infections in some countries despite long lockdown periods may be related to the social network structure and the underlying number and frequency of contacts.

An advantage of the network-augmented model, relative to the standard SIR model, is that the network model (hereafter NSIR) allows tracking (including via contact tracing) and distinguishing infections occurring through social contacts vs. at the population-level (unknown origin or community infections). The NSIR approach also allows modeling and analyzing richer behavioral responses, e.g., based on the disease state of an agent’s social contacts or deaths among one’s contacts, in addition to responses based on aggregate states. The main challenge to the network approach is the choice or calibration of the social network of contacts which is a key model input.

The social-network augmented NSIR model allows the researcher to specify and vary, via model and policy parameters, the relative rate of viral transmission within agents’ social network vs. the population and thus nests the standard SIR model as a special case. Since, unlike SIR, the NSIR model is simulated at the agent level it incorporates agent heterogeneity, via the agent’s position in the network by construction, but also extendable in other economically relevant dimensions. The NSIR model is solved via a stochastic Monte Carlo approach using the Gillespie algorithm ([15, 16]), a numerical method for generating statistically correct trajectories (possible solutions) of a stochastic system.

The proposed network-augmented model of COVID-19 is used to assess a broad set of simulated health and economic policies and behaviors, applying to all or subset of the population and including but not limited to:

(i) physical distancing—by varying the network structure (a reduction in the nodes’ degree / social contacts) and/or by varying the network-level vs. population-level mixing parameter.
(ii) testing and quarantine with or without contact tracing—by keeping track of and varying each agent’s network of allowed contacts in the simulation.
(iii) policy timing and duration—imposing or lifting health or economic policies at specific times or conditional on observed epidemic aggregates; both contiguous and intermittent policy interventions are considered.
(iv) endogenous behavioral responses by the agents (e.g., self-quarantine, avoiding contacts) based on observed infections or deaths among the agent’s contacts or in the population at large.

I am not an epidemiologist and all analysis and conclusions in this paper should be interpreted with the appropriate caveats. In addition, at the time of writing there is still a lot of uncertainty about the COVID-19 epidemiological parameter values and the policy outcomes are sensitive to that (robustness is explored). My objective is therefore primarily descriptive—to explore via simulations the implications, interactions and joint effects of epidemiological dynamics, social networks and policy or behavioral counterfactuals on health and economic outcomes.

My main findings are summarized as follows:

1. Viral transmission over a network-connected population can proceed slower and reach a lower peak than transmission via uniform/random contacts as assumed by standard SIR models. This is consistent with the findings of [17] using New York social interactions data. The resulting longer epidemic duration could imply larger overall economic costs, e.g., if accompanied by longer lockdown periods.
  In the NSIR model with network-based viral transmission:
2. Lockdown, quarantine and physical distancing policies which reduce the agents’ contacts are on average more effective in slowing down the viral transmission compared to in the SIR model with uniform mixing. Even partial lockdown or distancing can break or significantly reduce the transmission in the NSIR model by removing and isolating key network links, paths and nodes while these policies are less effective with uniform mixing. Large-scale and persistent testing and contact tracing are required to lower and flatten the infection rate curve. A low testing rate or a one-off mass testing campaign are not likely to be effective because of the relatively short serial interval of COVID-19.
3. If lifted early, lockdown or distancing policies mostly shift the infection peak into the future, with associated economic costs. Simulations show that one-, two- and four-month distancing policies starting from 0.5% infected share initially steadily reduce the number of active cases but could fail to contain the epidemic since a large number of susceptible non-immune agents remains at large. Mass vaccination, herd immunity (at the cost of many deaths), or a combination of mass-scale and persistent testing, contact tracing and enforced (self-)isolation appear the only reliable ways to stop the epidemic from reigniting if lockdown policies are lifted early. It may still take long to contain the COVID-19 epidemic when a vaccine is available. Unlike the virus, a vaccine does not replicate and spread on its own. Hence, a vaccine is only effective if introduced on a sufficiently large and/or optimally chosen subset of the population. For example, [18] show that a non-uniform (proportional to node degree) distribution of antidote in a network can control an epidemic while uniform antidote distribution cannot.
4. The epidemic dynamics are sensitive to policy timing and duration. The social-contacts network structure and infection time path (which nodes are infected when) also affects the spread of the epidemic unlike in the SIR model. Delayed lockdown or distancing policies or endogenous behavioral responses generate a multi-peaked infection rate over time, a form of ‘curve flattening’, but may have costlier economic consequences by prolonging the epidemic duration.
5. Intermittent (“on”, “off”, “on” again) lockdown or distancing policies and behaviors are demonstrated to be effective in flattening the infection curve. Intermittent policies can be politically easier to implement and enforce but may entail larger overall economic or healthcare costs.
6. Behavioral responses, through reducing the number or rate of social contacts based on observed infections, on aggregate or in one’s own network, can be a powerful and economically less costly alternative to mandated lockdowns but could induce a cyclical pattern of tightening and relaxation over a prolonged period.

## 2 The NSIR model

### 2.1 Setup

Consider a large population of *N* persons modeled as the nodes of a social network/graph *G*. The graph edges capture (regular) social interactions which are possible vectors of infection transmission. The assumed baseline network structure is an input of the model, however, health policies (e.g., lockdowns, quarantine, etc.), can be interpreted as (temporarily) changing the social network by eliminating edges (contacts). In addition to network-level contact, persons/nodes can also interact with any other node (connected or not) with rate/probability *p* ∈ [0, 1]. The limiting case *p* = 1 thus approximates the random mixing assumption in the standard SIR model.

Each node *i* = 1, …, *N* has an individual state *x*_*it*_ at time *t*. The basic model states are five: *S* for susceptible to the disease; *E* for exposed (infected but not yet infectious); *I* for infectious; *R* for recovered and *F* for dead. Additional states for ‘tested positive’ (known infected) or ‘in lockdown’ will be introduced in the policy simulations.

The NSIR model is initialized by randomly assigning #*I* ∈ (0, *N*) nodes to the infectious state, that is setting *x*_*i*0_ = *I*, *i* ∈ *I*_0_ and the rest of the nodes to the susceptible state, *x*_*j*0_ = *S* for *j* ∈ *S*_0_, where from now on *X*_*t*_ denotes the set of nodes/agents with state *x*_*it*_ = *X* at time *t*.

Conditional on current state *x*_*it*_, the next state *x*_*it*′_ for node *i* is determined as follows. The probability for any state transition not specified below, e.g., *S* to *I* or *E* to *R* is set to zero.

**(a) susceptible agents**

$$\begin{array}{c} x_{it^{\prime }}|\left(x_{it}=S\right)=\{\begin{array}{c} E\;\;\;\text{with}\;\text{prob.}\;p\beta \frac{I_{t}}{A_{t}}+\left(1-p\right)\beta \frac{\sum_{j\in C_{G}\left(i\right)}1_{x_{jt}=I}}{\#C_{G}\left(i\right)}\\ S\;\;\;\text{otherwise} \end{array} \end{array}$$ (1)

where *A*_*t*_ denotes the number of active agents at *t* (for example, all living agents, *A*_*t*_ = *N* − *F*_*t*_) and where *C*_*G*_(*i*), with dimensionality (node degree) #*C*_*G*_(*i*), denotes the set of contacts / edges of node *i* in the social graph *G*. The notation **1**_*x*_*jt*_ = *I*_ is an indicator function which equals 1 if *x*_*jt*_ = *I* and zero otherwise. The parameter *β* captures the contact rate and infection rate conditional on contact with an infectious person. The expressions $\frac{I_{t}}{A_{t}}$ and $\frac{\sum_{j\in C_{G}\left(i\right)}1_{x_{jt}=I}}{\#C_{G}\left(i\right)}$ are the probabilities that the contact is infectious, in the population or in one’s social network, respectively.

The first term (multiplied by *p*) in (1) captures the rate of infection from contact with an infectious person in the population at large (e.g., public transit, shopping, etc.) This term corresponds to the uniform mixing (random meeting) transmission vector in the standard SIR model. The second term in (1) (multiplied by 1 − *p*) captures viral transmission that occurs because of an existing infection(s) among *i*’s social contacts in *G*, the set *C*_*G*_(*i*). In Section 3 I show how (1) can be modified to include testing and quarantine/isolation.

**(b) exposed agents**

$$\begin{array}{c} x_{it^{\prime }}|\left(x_{it}=E\right)=\{\begin{array}{c} I\;\;\;\text{with}\;\text{prob.}\;\sigma \\ E\;\;\;\text{with}\;\text{prob.}\;1-\sigma \end{array} \end{array}$$ (2)

The transition from the exposed to the infectious state happens at rate *σ* set to match the disease’s incubation period.

**(c) infectious agents**

$$\begin{array}{c} x_{it^{\prime }}|\left(x_{it}=I\right)=\{\begin{array}{c} R\;\;\;\text{with}\;\text{prob.}\;\gamma \\ F\;\;\;\text{with}\;\text{prob.}\;\mu \\ I\;\;\;\text{with}\;\text{prob.}\;1-\gamma -\mu \end{array} \end{array}$$ (3)

The expected recovery rate is *γ*. The fatality rate conditional on being infected is *μ*.

**(d) recovered agents and deaths**

$$\begin{array}{cc} x_{it^{\prime }}|(x_{it} & =R)=R\;\;\;\text{with}\;\text{prob.}\;\text{1}\\ x_{it^{\prime }}|(x_{it} & =F)=F\;\;\;\text{with}\;\text{prob.}\;\text{1} \end{array}$$ (4)

Death (state *F*) and recovery (state *R*) are absorbing states. Possible transition from state *R* back to the susceptible state *S* is ruled out in the simulations but is very easy to incorporate via an additional parameter. Base population birth or death rates can be also modeled but I abstract from this here.

There are two main groups of parameters in the model. The parameters *β* (infectiousness), *σ* (incubation period), *γ* (survivability) and *μ* (mortality) are assumed biologically fixed in the baseline simulations. It is computationally feasible to allow state-based mortality rate, *μ*(*I*_*t*_) (for example, because of exceeding hospital capacity) as in [3]. In contrast, the parameter *p* and the social network structure *G* on which agents interact are interpreted as socioeconomic variables affected by policy or behavioral responses. In Section 3 I introduce additional policy parameters and graphs to model testing, contact tracing, lockdowns, distancing and quarantine.

### 2.2 Simulation

The model is initialized by choosing the initial number of infectious nodes (*I*_0_), with the rest of the *N* nodes set in susceptible *S* state. A baseline network graph, *G* of size *N* is also chosen (see Section 4.1 for details and Section 6 for alternative specifications and robustness).

Model time evolves stochastically from *t* to *t*′ = *t* + *τ*, by having the time index *t* increased by the amount *τ* computed from the state-transition probabilities in (1), (3) and (2) using Gillespie’s algorithm, see [16]. One unit of time equals one day. Only the state of a single randomly selected node is modified at each time increment *τ*. All other nodes retain their previous states. The Matlab codes used in this paper (available at the author’s website) draw on and significantly extend publicly shared Python code by Ryan McGee, see https://github.com/ryansmcgee.

Formally, for each *t*, the Gillespie algorithm executes the following steps:

(i) draw two random scalars *r*_1_ and *r*_2_ from the uniform distribution on (0, 1)
(ii) compute the total probability of any node changing to a new state using (1), (3) and (2), call it Π
(iii) use *r*_1_ to draw the time interval *τ* until the next state change event as $\tau =\frac{1}{\Pi }\ln\left(\frac{1}{r_{1}}\right)$, using that the state change time interval is exponentially distributed with mean $\frac{1}{\Pi }$. Call *t*′ = *t* + *τ*.
(iv) use the draw *r*_2_ to select one of all positive-probability state transitions, *x*_*lt*_ to *x*_*lt*′_ with *x*_*lt*′_ ≠ *x*_*lt*_ implied by (1), (3) and (2), with corresponding transitioning node *l* ∈ {1,..*N*}. The chance of selecting a specific transition is proportional to its probability.
(v) perform the state transition from Step (iii) by updating node *l*’s state and keeping all other nodes’ states the same as at *t*
(vi) forward model time to *t*′ = *t* + *τ* and go back to Step (i)

Model time is forwarded by larger intervals when transition events are relatively rare (e.g., few initial infections or low values of *β*, *γ*, *σ* and *μ*) and by small intervals when transition events are frequent (many nodes with high total transition probability around the same *t*). The total rates of susceptible, exposed, infectious, recovered and dead agents are calculated at any model time *t* by adding up over *i* the individual states *x*_*it*_. For example, the total number of infectious persons is *I*_*t*_ = ∑_*i*_ **1**_*x*_*it*_ = *I*_.

In sum, the NSIR model allows keeping track of and simulating:

(i) each node’s individual disease state (*S*, *E*, *I*, *R* or *F*) over time
(ii) the evolution of aggregates over time, including total infections, total recoveries, total deaths, etc.
(iii) daily changes in the aggregates (over model time intervals with length *Δt* = 1)
(iv) state transitions over time and over the social network *G* by using *G*’s adjacency matrix; for example, this allows tracking the states of nodes with large vs. small number of contacts (edges in *G*) and comparing and tracing the spread of the disease via social-contacts vectors vs. at the population level (random mixing).

### 2.3 NSIR vs. SIR reproduction dynamics

#### 2.3.1 Basic and effective reproduction numbers

In epidemiology the basic reproduction number, $R_{0}$ is the expected number of cases that the first infected person generates, when all other agents are susceptible but not yet infected. In the standard S(E)IR model without social network component,

$$\begin{array}{c} \frac{d(E_{t}+I_{t})}{dt}=\beta \frac{I_{t}S_{t}}{N}-rI_{t}=rI_{t}\left(\frac{\beta }{r}s_{t}-1\right) \end{array}$$

where *r* = *γ* + *μ* is the removal rate and $s_{t}\equiv \frac{S_{t}}{A_{t}}$ is the fraction of susceptible agents at time *t* out of all active agents *A*_*t*_. Early on, or with few deaths, *A*_*t*_ ≃ *N* yielding an effective reproduction number

$$\begin{array}{c} R_{t}^{SIR}=\frac{\beta }{r}\frac{S_{t}}{N}\text{.} \end{array}$$ (5)

Evaluating at *S*_0_ ≃ *N* gives the familiar SIR $R_{0}$ value, $R_{0}^{SIR}=\frac{\beta }{r}$. If $\frac{\beta }{r}>1$ the epidemic grows (if unchecked) as long as there is a sufficiently large fraction of susceptibles, $s_{t}=\frac{S_{t}}{N}>\frac{1}{R_{0}^{SIR}}$. In contrast, if $R_{0}^{SIR}<1$ the epidemic would die out on its own.

I define the effective reproduction number $R_{t}$ in the NSIR model analogously. Define

$$\begin{array}{c} \sigma_{it}\left(G\right)\equiv \frac{\sum_{j\in C_{G}\left(i\right)}1_{x_{jt}=I}}{\#C_{G}\left(i\right)}\text{,} \end{array}$$

corresponding to the agent *i*’s probability of infection from one of her social contacts in graph *G* at time *t*. Using (1), in the (no-intervention) NSIR model we have,

$$\begin{array}{cc} \frac{d(I_{t}+E_{t})}{dt} & =p\beta I_{t}s_{t}+\left(1-p\right)\beta \sum_{i\in S_{t}}\sigma_{it}\left(G\right)-rI_{t}=\\ & =rI_{t}(p\frac{\beta }{r}s_{t}+\left(1-p\right)\frac{\beta }{r}\frac{S_{t}}{I_{t}}\frac{\sum_{i\in S_{t}}\sigma_{it}\left(G\right)}{S_{t}}-1) \end{array}$$

The number of infected agents (exposed plus infectious) would grow if the expression in the brackets is positive. Hence, for $s_{t}≃\frac{S_{t}}{N}$, define the NSIR model effective reproduction number $R_{t}^{NSIR}$ as

$$\begin{array}{c} \frac{\beta }{r}\left[p\frac{S_{t}}{N}+\left(1-p\right)\frac{S_{t}}{I_{t}}\frac{\sum_{i\in S_{t}}\sigma_{it}\left(G\right)}{S_{t}}\right] \end{array}$$ (6)

At *p* = 1 this expression equals $R_{t}^{SIR}$ but in general, including at *t* = 0, the NSIR model reproduction number $R_{t}^{NSIR}$ differs from $R_{t}^{SIR}$ and depends on the graph *G*. For example, [9] emphasize the importance of the ratio between the second and first moment of the degree distribution for the infection growth rate.

#### 2.3.2 Population vs. network transmission

I next compare the reproduction numbers for the SIR model (*p* = 1) and the network-only transmission NSIR model (*p* = 0) for given values of *I*_*t*_ and *S*_*t*_. Using (6), for *p* = 0 we have

$$\begin{array}{c} R_{t}^{NSIR}=\frac{\beta S_{t}}{rI_{t}}\frac{\sum_{i\in S_{t}}\sigma_{it}\left(G\right)}{S_{t}} \end{array}$$ (7)

where

$$\begin{array}{c} \frac{\sum_{i\in S_{t}}\sigma_{it}\left(G\right)}{S_{t}}\equiv {\bar{\sigma }}_{t}\left(G\right) \end{array}$$

is the average chance of infection across all susceptible nodes *i* ∈ *S*_*t*_ at time *t*, given the set $I_{t}$ of infectious agents *i* with *x*_*it*_ = *I*. Comparing (5) and (7), observe that

$$\begin{array}{c} R_{t}^{NSIR}⋛R_{t}^{SIR}\Leftrightarrow {\bar{\sigma }}_{t}\left(G\right)⋛\frac{I_{t}}{N} \end{array}$$ (8)

Intuitively, the standard SIR model assumes a *uniform* chance of infection for each susceptible agent which is proportional to the population infection rate $\frac{I_{t}}{N}$. In contrast, in the network-augmented NSIR model an individual’s chance of infection is *heterogeneous* and is a function of the social network *G*. The average time-*t* infection probability in the network, ${\bar{\sigma }}_{t}\left(G\right)$ determines the reproduction number $R_{t}^{NSIR}$. For example, consider the first infection, *I*_0_ = 1 of some agent *i*_0_, at which $R_{0}^{SIR}≃\frac{\beta }{r}$. In contrast, the value of the NSIR effective reproduction number $R_{0}^{NSIR}$ would depend on ${\bar{\sigma }}_{0}\left(G\right)$, which is a function of the graph *G* and of which node was initially infected (path dependence).

Using (5) and (7), it is clear that the growth rate of the disease would differ in general in the SIR (*p* = 1) vs. NSIR model (*p* = 0) and the counts *I*_*t*_ and *S*_*t*_ would generally differ in calendar time *t*. Thus, to proceed with the comparison, I compare the SIR vs. NSIR reproduction numbers for the same cumulative infection count *m* for several example graphs *G*. To avoid potential confusion with calendar time, for the rest of this section I will use *I*(*m*) and *S*(*m*) to denote the number of infectious and susceptible agents at the time of the *m*-th infection.

**Result 1**: *For the same infection count, the SIR model effective reproduction number equals that of the NSIR model on a complete graph*.

Proof sketch: Suppose *G* is a complete graph (each node is connected to all other nodes) and *N* is large. The first infection, *m* = 1 yields *N* − 1 susceptible agents with average chance of infection $\sigma_{i(1)}=\frac{1}{N-1}$ each and so $\frac{\sum_{i\in S(1)}\sigma_{i(1)}}{S(1)}≃\frac{1}{N}$. The second infection yields *S*(2) = *N* − 2 with $\sigma_{i(2)}=\frac{I(2)}{N-1}$ each and so $\frac{\sum_{i\in S(2)}\sigma_{i(2)}}{S(2)}≃\frac{I(2)}{N}$. Continuing in the same way, for the *m*-th infection there are *S*(*m*) susceptible agents with $\sigma_{i(m)}=\frac{I(m)}{N-1}$ each and so $\frac{\sum_{i\in S(m)}\sigma_{i(m)}}{S(m)}≃\frac{I(m)}{N}$—the SIR and NSIR reproduction numbers are equal. If a formerly infectious node recovers or dies in the process, then *I*(*m*) is reduced in both SIR and NSIR.

*Example 1. Regular graph*. Suppose *G* is a connected regular graph in which each node has degree *K* ∈ [2, *N*). After the first infection, *K* susceptible agents have infection probability $\sigma_{i(1)}=\frac{1}{K}$ while for the rest *σ*_*i*(1)_ = 0, yielding ∑_*i*∈*S*(1)_ *σ*_*i*(1)_ = 1. Then, since *S*(1)≃*N*, we obtain $\bar{\sigma }\left(1\right)≃\frac{1}{N}=\frac{I(1)}{N}$, that is, $R_{0}^{NSIR}=R_{0}^{SIR}$. Consider now the second infection, of some node *j*_1_ which by construction is *one of the contacts* of the first infected node *j*_0_. Hence only *K* − 1 susceptible agents could be infected by *j*_0_ and *j*_1_ each. If a node *h* is connected to both *j*_0_ and *j*_1_ we can think of splitting the total probability *σ*_*h*_ as 1/2 coming from each. Thus, $\sum_{i\in S(2)}\sigma_{i(2)}=I\left(2\right)\left(K-1\right)\frac{1}{K}$ and so ${\bar{\sigma }}_{1}\left(G\right)≃\frac{I(2)}{N}\frac{K-1}{K}$ which is strictly less than $\frac{I(2)}{N}$ and so $R^{NSIR}<R^{SIR}$. A similar argument applies for further infections. As a result, for the same infection count *m*, the average chance of infection in a regular-graph NSIR model is lower than the population infection rate $\frac{I(m)}{N}$ in the SIR model.

*Example 2. Ring graph*. Suppose *G* is a connected ring graph, such that each node *i* = 1,..*N* is only connected to two nodes, *i* − 1 and *i* + 1 (where node index 0 maps to *N* and *N* + 1 maps to 1). For the first infection $\bar{\sigma }\left(1\right)≃\frac{I(1)}{N}$ and $R_{0}^{NSIR}=R_{0}^{SIR}$, as in Example 1. By construction, any subsequent infectious node must be a contact of a previously infectious node, thus at any time the set of infectious and recovered/dead nodes, I is contiguous (consists of nodes that are neighbors on an arc *j*, *j* + 1, ..*j* + *l*). Hence, at each next infection count step, *m* = 2, 3, … there are only at most 2 susceptible nodes in a ring graph (the outside neighbors of the set I) which have positive probability of infection *σ*_*i*_ = 1/2. For example, if the set I consists of nodes 2,3,4,5 positioned in order on the ring graph, the end-nodes are 2 and 5. There would be 1 or 0 susceptible nodes that can be infected if an end-node of I has already recovered/died. For the rest of the susceptible nodes *σ*_*i*_ = 0, since in a ring graph they are not connected to any nodes in I. Therefore, $\bar{\sigma }\left(m\right)\leq \frac{1}{S(m)}$ which is (much) smaller than $\frac{I(m)}{N}$ for *m* small. Thus, using (8) we obtain $R^{NSIR}<R^{SIR}$. If, as time progresses, both end-nodes of the set I become recovered/dead before a new node is infected (this can occur with positive probability) then the epidemic dies out in the network model but not necessarily in the SIR model (if interior nodes in I remain infectious).

*Example 3. Star graph*. Suppose *G* is a star graph in which a single node *j* is connected to all *N* − 1 other nodes and there are no other edges. If the first infected node is *j* then $\bar{\sigma }\left(1\right)≃1>\frac{I(1)}{N}=\frac{1}{N}$ and so $R_{0}^{NSIR}>R_{0}^{SIR}$. If the first infected note is instead one of the ‘rays’, then $\bar{\sigma }\left(1\right)≃\frac{1}{N}$ but since the second infected node is necessarily *j*, we obtain again $\bar{\sigma }\left(2\right)≃1>\frac{I(2)}{N}$.

These examples show that the graph structure and the network node path followed by the infection over time (the subgraph of infected nodes) are key determinants of the effective reproduction number and hence infection growth (see also Fig 11 in Section 4.5). Degree heterogeneity combined with high-degree nodes infected early on could raise the NSIR reproduction number above the SIR value (see also [9]), while graphs in which the degree distribution is relatively homogeneous are likely to have lower reproduction rates than in the SIR model. Degree heterogeneity could also be critical in determining policy outcomes, e.g., the infection reaching a superspreader can accelerate or re-ignite the epidemic—see Section 4.4 and Fig A and E in the S1 Appendix for further discussion and examples.

#### 2.3.3 Local vs. bridge links

The ring graph Example 2 suggests that policies and behavioral responses (for example, related to large gatherings, travel, border closures) which restrict the epidemic on a smaller or localized set of nodes can have significant impact on the effective reproduction number and therefore on the overall infection count, hospitalizations, deaths and related economic costs.

**Fig 1:**
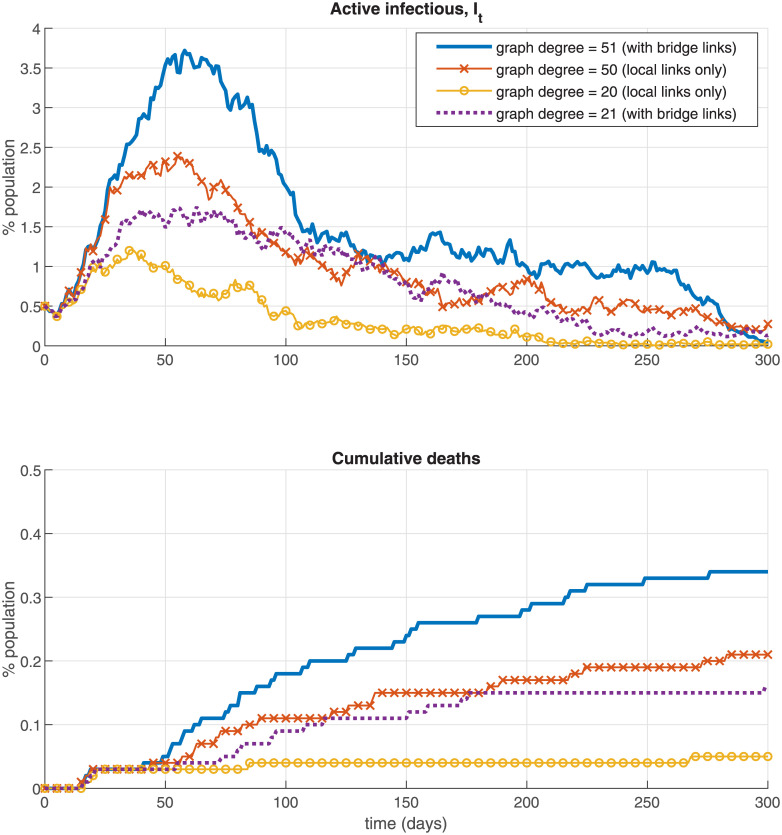
Regular graphs example—Local vs. bridge links.

I illustrate this idea further via simulations on Fig 1 which plots the infection rate and total death rate over time in the NSIR model with a *regular graph* *G* with degree *d* constructed in a specific way (the Figure uses *p* = 0 and the baseline model parameters in Table 3 and no interventions). Fig 1 is just an example, for this section only (the main simulation results use the graphs described in Section 4.1).

The regular graph in Fig 1 when *d* is *even* (*d* = 20 or 50) is constructed by setting all nodes on a circle and then each node *i* is connected to the *d*/2 nodes immediately before (*i* − 1, …*i* − *d*/2) and immediately after it (*i* + 1, …, *i* + *d*/2). The ring graph in Example 2 corresponds to the case *d* = 2. Thus, for *d* even, each node is only connected to other nodes in its locality, that is, an infectious node can only infect susceptible nodes near it (up to distance *d*/2).

In contrast, when *d* is *odd*(*d* = 21 or 51 in Fig 1) each node of *G* is connected to the (*d* − 1)/2 nodes immediately before and after it (analogously to the *d* even case) but, in addition, to node *i* + *N*/2, that is, the node “across” from *i* on the graph circle. This means that each infectious node now has a positive probability of spreading the virus to a new, “far” area of the graph *G*—a “bridge” link. Fig 1 shows that a minor difference in the graph degree (20 vs. 21 or 50 vs. 51) can have a significant effect on the infection and death rates. Specifically, when bridge links are present in the social contacts graph *G* (the odd-degree cases *d* = 21 and 51) the share of active infections and total deaths can be 2 or 3 times larger than in the ‘local contacts only’ cases (even-degree, *d* = 20 and 50). In contrast, there is almost no difference between the simulations using *d* = 18 vs. 20 or 48 vs. 50 (not displayed on the Figure). The conclusion is that interventions that aim at restricting the epidemic on a local level and eliminate bridge contacts (e.g., air travel) can be effective in suppressing the epidemic.

**Fig 2:**
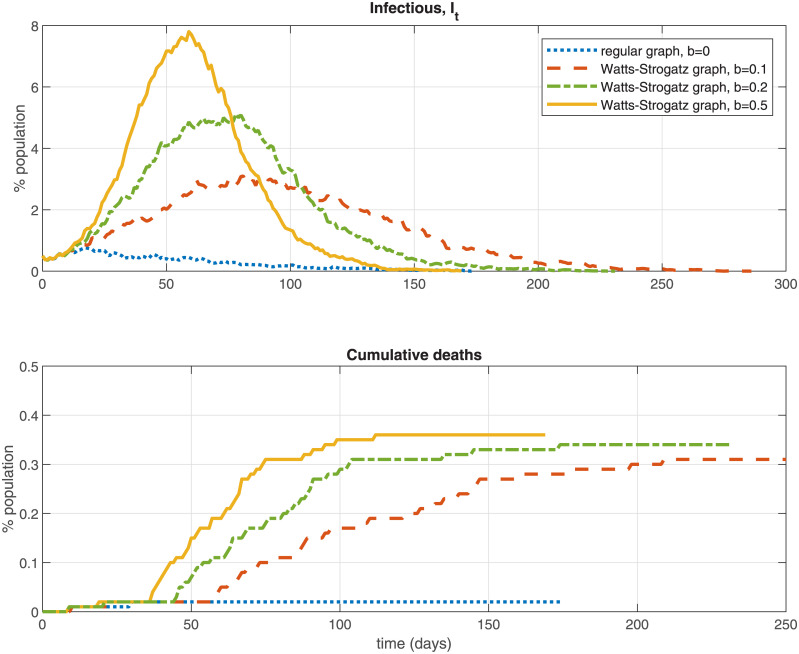
Watts-Strogatz graphs example.

On Fig 2 I explore further the role of the network structure for viral transmission dynamics and the infection rate over time. The Figure computes the infection curve for a series of graphs, starting with a regular (ring-style) graph in which each node is only connected to nearby nodes (the dotted line) and comparing it to three Watts-Strogatz graphs with the same median degree 12 and number of nodes (*N* = 10, 000) but with different values for the parameter *b* that governs the probability of re-wiring an edge to a new node. Larger values of *b* correspond to more re-wiring. i.e., adding more bridging contacts with non-local nodes. The results show that an increased number of cross-links added to the contacts graph can raise the infection peak, total infections, and cumulative deaths significantly (e.g., the peak infection rate is 0.8% in the regular graph, *b* = 0 vs. 7.8% in the Watts-Strogatz graph with *b* = 0.5), consistent with the theoretical discussion above.

## 3 Policies and scenarios

The NSIR model can be used or extended to incorporate a wide variety of health and socioeconomic policies and scenarios related to mitigating or failing to control the spread of the disease.

**1. Herd immunity**—simulating the NSIR model without any policy intervention or behavioral response.

**2. Testing**

Testing is modeled by introducing an additional state *P* (“tested positive”). Assume for simplicity that only infectious (state *I*) agents can test positive. The transition probabilities in (3) are modified to:

$$\begin{array}{c} x_{it^{\prime }}|\left(x_{it}=I\right)=\{\begin{array}{c} R\;\;\;\text{with}\;\text{prob.}\;\gamma \\ P\;\;\;\text{with}\;\text{prob.}\;\theta \\ F\;\;\;\text{with}\;\text{prob.}\;\mu \\ I\;\;\;\text{with}\;\text{prob.}\;1-\gamma -\mu -\theta \end{array} \end{array}$$ (9)

where *θ* is the fraction of currently infectious agents tested per unit of time. Agents with *x*_*it*_ ≠ *I* are assumed to always test negative (allowing the possibility of positive test for state *E* is simple). Keeping track of “tested negative” agents can be easily incorporated too (e.g., to keep track of testing costs or testing coverage over time).

The transition probabilities for the agents who have tested positive (state *P*) are:

$$\begin{array}{c} x_{it^{\prime }}|\left(x_{it}=P\right)=\{\begin{array}{c} R\;\;\;\text{with}\;\text{prob.}\;\gamma_{P}\\ F\;\;\;\text{with}\;\text{prob.}\;\mu_{P}\\ P\;\;\;\text{with}\;\text{prob.}\;1-\gamma_{P}-\mu_{P} \end{array} \end{array}$$ (10)

where the recovery and fatality parameters *γ*_*P*_ and *μ*_*P*_ can be the same of different than *γ* and *μ* in (3) and (9).

The agents who test positive, *x*_*it*_ = *P* are assumed to be isolated or in (self-)quarantine and not mixing with others in the population; that is, *A*_*t*_ = *N* − *F*_*t*_ − *P*_*t*_ in (1). However, the *P* agents could still infect contacts in their immediate social network *Q*, defined as a sub-graph of *G* with the same nodes but fewer edges per node (see more details below). That is, the second term in (1) is modified to

$$\begin{array}{c} \left(1-p\right)\beta (\frac{\sum_{j\in C_{G}\left(i\right)}1_{x_{jt}=I}\;+\;\sum_{j\in C_{Q}\left(i\right)}1_{x_{jt}=P}}{\#C_{G}\left(i\right)}) \end{array}$$

**3**. **Contact tracing**

The network aspect of the NSIR model is well-suited to study *contact tracing*, that is, following up, identifying and isolating the contacts of agents who have tested positive. Contact tracing is modeled by adding a parameter *ϕ* and a new term in (9), interpreted as the additional probability of identifying an agent *i* as infectious (and moving *i* to state *P*) for each of *i*’s contacts *j* who have tested positive.

$$\begin{array}{c} x_{it^{\prime }}|\left(x_{it}=I\right)=P\;\;\;\text{with}\;\text{prob.}\;\min\left\{1,\theta +\phi \sum_{j\in C_{G}\left(i\right)}1_{x_{jt}=P}\right\} \end{array}$$

**4. Distancing and quarantine**—physical (social) distancing can be incorporated in two complementary ways, both of which are explored in the simulations in Section 4. The first way of modeling distancing is by decreasing the value of the parameter *p*. This corresponds to setting a lower rate of global (population level) interactions and higher rate of local (network-level) interactions in (1). A second way of modeling distancing is by varying the network structure, that is, replacing the baseline social network *G* with another network *D* which is a sub-graph of *G* with fewer edges connected to each node (lower degree).

Quarantine, an extreme form of distancing is modeled by setting *p* = 0 and assuming a very small number or zero social contacts for each quarantined node (their narrow social graph *Q*).

**5. Lockdown**—assume that fraction *λ* ∈ (0, 1) of all agents are locked down and only the remaining fraction 1 − *λ* of agents interact, similar to [3]. This is done by introducing an indicator variable (‘locked down’, *L* or ‘not locked down’, ¬*L*) for each node *i* and modifying (1) as follows:

$$\begin{array}{cc} Prob(x_{it^{\prime }} & =E\;|\;x_{it}=S\wedge \neg L)=p\beta \frac{\left(1-\lambda \right)I_{t}}{\left(1-\lambda \right)N-F_{t}}+\left(1-p\right)\beta \frac{\sum_{j\in C_{G}\left(i\right)}1_{x_{jt}=I\wedge \neg L}}{\#C_{G}\left(i\right)}\\ Prob(x_{it^{\prime }} & =E\;|\;x_{it}=S\wedge L)=\beta \frac{\sum_{j\in C_{Q}\left(i\right)}1_{x_{jt}=I}}{\#C_{Q}\left(i\right)} \end{array}$$ (11)

The first term in (11) assumes that the probability of contact with a random person remains unchanged for the agents not in lockdown (e.g., interact with others at work). An alternative would be to assume reduced frequency of contacts, for example, $p\beta \left(1-\lambda \right)\frac{\left(1-\lambda \right)I_{t}}{\left(1-\lambda \right)N-F_{t}}$. Locked down agents, the second line in (11), are assumed to be exposed only to their narrow social network *Q*, a sub-graph of *G* (e.g., close family) with the same number of nodes but fewer edges per node.

**6. Behavioral responses**

The NSIR model allows incorporating a rich set of endogenous behavioral responses to the epidemic. The agents can decide to reduce the number or rate of their contacts, based on observable information or individual cost-benefit calculations (see also [19, 20] or [21] in non-network models). Specifically, suppose *p* < 1 and define the following social-contact graphs: *E*_0_ = *G* and *E*_*k*_ ⊂ *E*_*k*−1_ for *k* = 1, …*M*, where ⊂ *X* denotes a sub-graph of *X* with the same nodes but fewer edges/contacts per node. For example, if *M* = 2 we can think of *E*_0_ = *G* as the “normal times” social network; *E*_1_ ⊂ *G* as a “reduced contacts” network (e.g., work and necessity shopping); and *E*_2_ ⊂ *E*_1_ as a “close family” network.

In the simulations in Section 4.3 each agent is assumed to switch to a more restricted (lower-degree) network, based on the observed infection rate in the population (aggregate-level information) or, alternatively, based on positive case(s) in their own social network *C*_*G*_(*i*) (individual-level information).

Each policy or behavioral scenario 1 through 6 can be imposed or lifted in the simulations at a pre-specified model time *t* ∈ (0, *t*_max_) or conditional on reaching a specific aggregate state value (e.g., number of positive tests or deaths per day, total positive cases, etc.). I investigate a range of scenarios in the following sections.

## 4 Simulation results

### 4.1 Baseline parameters and initial conditions

Table 1 reports the baseline parameter values used in the model simulations. The baseline expected removal rate *r* is set to 0.2 which corresponds to a 5-day average period of infectiousness ([22, 23]), following a 5.2-day average exposed stage duration (the parameter *σ*). I also explore a longer infectiousness period, *r* = 0.1 in the robustness checks in Section 6. The baseline infection fatality rate (IFR) is set to 0.37% using Streeck et al.’s German randomized study, [24]. An 0.66% estimated IFR with Wuhan data, (e.g., [25]) and 1% IFR are also explored in the robustness Section 6.2. The IFR value is important for the death total but, since *μ* is small and death is an absorbing state, it otherwise changes very little the infection rate dynamics (see Fig 13 in Section 6.2).

**Table 1:** Baseline parameter values and initial conditions.

**Parameter**	**Value**	**Description**	**Source, etc.**
*r*	0.2	removal rate	Anderson et al.; 5-day avg. duration post incubation
*μ*	0.0037*r*	mortality rate	0.37% IFR, Streeck et al.
*γ*	*r* − μ	recovery rate	based on *r* and *μ*
*β*	0.5	infectivity rate	approx. 3-day initial doubling time/implied *R*_0_ = 2.5
*σ*	1/5.2	incubation, days^−1^	Wang et al.; median incubation period 5.2 days
*θ*	2%, 5%	mass testing rate	hypothetical / assumed
*ϕ*	10%	contact tracing rate	hypothetical / assumed
**Init. condition**	**Value**	**Description**	
*N*	10,000	population / network size	
*I*_0_	1, 50	initial number of infections	
*t*_max_	200, 500	maximum simulation duration in days	
*G*	n.a.	modified Barabasi-Albert graph with median degree 10 (min = 0, max = 200)	
*Q*	n.a.	modified Barabasi-Albert graph with median degree 1 (min = 0, max = 14)	

The value of the COVID-19 infectiousness *β* is calibrated to fit the observed approximately three-day early doubling time of the disease (e.g., [26]) and/or a basic reproduction number *R*_0_ of 2.5. The calibrated parameters are actual as of early May 2020. Versions of most figures using alternative parameter values, corresponding to slower infection growth and higher IFR (*r* = 1/16, *μ* = .0066*r* and *β* = .156) are available on request. In the simulations below I set the recovery and mortality rates for positive agents (in state *P*) to be the same as the baseline values, *γ*_*P*_ = *γ* and *μ*_*P*_ = *μ*.

A key ingredient of the NSIR model is the social contacts graph *G*. I use as baseline a *modified* (pruned) version of a Barabasi-Albert (B-A) graph, constructed starting from a Barabasi-Albert graph with 9-edge preferentially attached nodes and then randomly removing a fraction of edges to generate node degrees lower than 9. The resulting social contacts graph *G* has median degree 10 and mean degree 12.6. The reason for choosing this baseline graph is that neither the standard scale-free B-A graph nor the standard small-world Watts-Strogatz (W-S) graph match well certain network properties documented in actual COVID-19 or other epidemic transmission networks (see [14], [13], [27–29]), namely broad degree heterogeneity and long/heavy right tail (superspreaders).

Standard B-A graphs match well the breadth and long right tail of the degree distribution (allow for superspreaders) but truncate the minimum node degree to a value close to the median, essentially ruling out nodes with few contacts. Watts-Strogatz graphs capture well short paths and local clustering realistic in many social networks but feature a relatively homogeneous degree distribution (all nodes have similar degree) and lack a long right tail, that is, they exhibit insufficient heterogeneity and broadness in the number of contacts and lack of superspreaders. The modified B-A graph *G* used in this paper matches both the broad heterogeneity of the degree distribution, including nodes with zero or low degree, and a long/heavy right tail—features also emphasized in the theoretical analysis in Section 2.3. Robustness simulations with W-S graphs are reported in Fig 2 and Section 6 showing that the main patterns and results remain robust.

Fig B in the S1 Appendix compares the degree distributions of the baseline graph *G* with that of a standard Albert-Barabasi graph (the input graph used in the edge removal procedure described in Section 4.1) and a standard Watts-Strogatz graph with mean degree 12. Fig C in S1 Appendix depicts the degree distribution of the baseline graph *G* and the closed-contacts graph *Q* constructed in the same way as *G* but with larger number of removed edges.

### 4.2 Results

I report simulation results from different policy and behavioral scenarios in the NSIR model. All graphs in this Section show sample simulation paths (one possible time path of the dynamic system), however, the same pseudo-random number sequences are used so the graphs are comparable across the scenarios. Summary Table 3 in Section 6.1 and Tables 1 and 2 in S1 Appendix in report average values from 100 simulations each, using the same parameters but 100 different pseudo-random number sequences (these sequences are held constant across the different parameter/policy specifications for comparability).

#### 4.2.1 No intervention vs. testing and contact tracing

**Fig 3:**
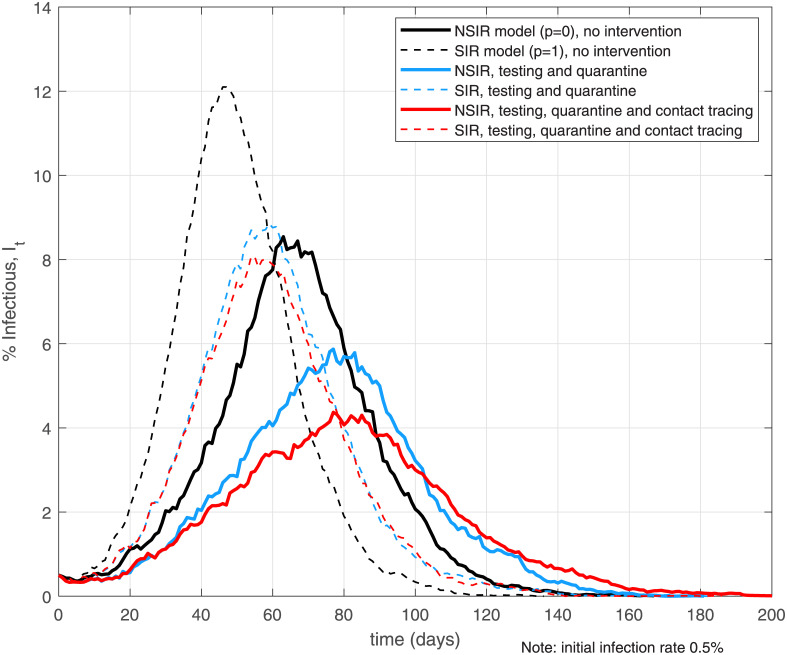
No intervention, mass testing, quarantine and contact tracing. Notes: the black lines assume no policy interventions or behavioral responses. The light blue lines assume mass testing rate *θ* = 0.05 and putting positive cases (state *P*) in quarantine, network *Q*. The red lines add contact tracing at rate *ϕ* = 0.1 to the testing and quarantine setting.

Fig 3 plots simulation paths in the NSIR setting with network transmission (p = 0, the solid lines) and the SIR setting with uniform transmission (p = 1, the dashed lines) for three basic scenarios—no intervention, testing and quarantine, and testing, quarantine and contact tracing. The Figure plots the percent of infectious nodes (in state *I*) over time across the different scenarios and settings.

The black lines on Fig 3 assume epidemic dynamics absent any intervention and/or behavioral responses (herd immunity). The blue lines use a testing rate *θ* = 0.05, that is, 5% of the currently infectious agents are assumed to be detected per day. i.e., a 25% average total chance of positive test for an infectious agent. This hypothetical testing rate is much higher compared to current daily testing rates in the world so these results should be interpreted as a “mass testing” counterfactual. The simulations assume *persistent* testing at rate *θ*, not a one-off testing campaign. A one-off campaign would only detect fraction *θ* of the *currently* infectious agents and thus is much less effective. The agents who test positive (enter state *P*) are assumed to be quarantined and interact only on a close-contacts social network *Q* (see Section 3). The red lines on Fig 3 assume contact tracing at rate *ϕ* = 0.1 added to the mass testing and quarantine. A 0.1 contact tracing rate means 10% additional daily probability of an agent testing positive for each of the agent’s contacts who have tested positive. Recently recovered or dead contacts can be easily incorporated.

The simulation results depicted on Fig 3 confirm that testing and contact tracing slow the infection growth rate and reduce the total infected, peak infected and deaths in both main model settings (see also Table 3), however, these reductions are larger in the NSIR, *p* = 0 setting. Tables 1 and 2 in S1 Appendix further quantify these results by reporting averages over 100 simulations. The larger policy impact in the network setting is especially pronounced for contact tracing (Table 2 in S1 Appendix)—the decrease in total infections or deaths in the NSIR setting can be double that in the SIR setting, relative to the respective no-tracing baseline. Intuitively, testing and contact tracing in the network setting (p = 0) can isolate high-degree infectious nodes (superspreaders) early and thus reduces the infection rate by a larger amount—this effect is absent in the uniform-mixing SIR, p = 1 model setting, as previously discussed in Section 2.3.

Tables 1 and 2 in S1 Appendix also show that a 0.1% testing rate has very small effect on the infection aggregates, except a 3.9% reduction in deaths in the p = 0 setting. To make a serious dent in overall infections and deaths, very intensive testing and quarantine is required (*θ* = 10%), with the downside of a significantly prolonged (+41%) epidemic duration. Table 2 in S1 Appendix further shows that, holding the testing rate constant, increasing the intensity of contact tracing yields additional large reductions in total infections, deaths and the infection peak with this effect being stronger in the NSIR, p = 0 model.

#### 4.2.2 Distancing policies

In this Section I simulate several physical distancing policies in the NSIR model with network-level transmission, *p* = 0. The duration and timing of the policy is represented by the shaded area on the graphs. During the distancing period it is assumed that all agents’ interactions occur on the truncated social network *Q* defined as a sub-graph of the original social network *G* whereby each node’s degree is randomly scaled down (an agent’s contacts are reduced by 10 times on average). To explore different policy lengths and timings all simulations are initialized with 0.5% infectious agents; the timelines on Figs 2 and 3 and Table 3 are relative to that moment. Shorter or less strict policies can be effective at lower initial infection rates.

**Fig 4:**
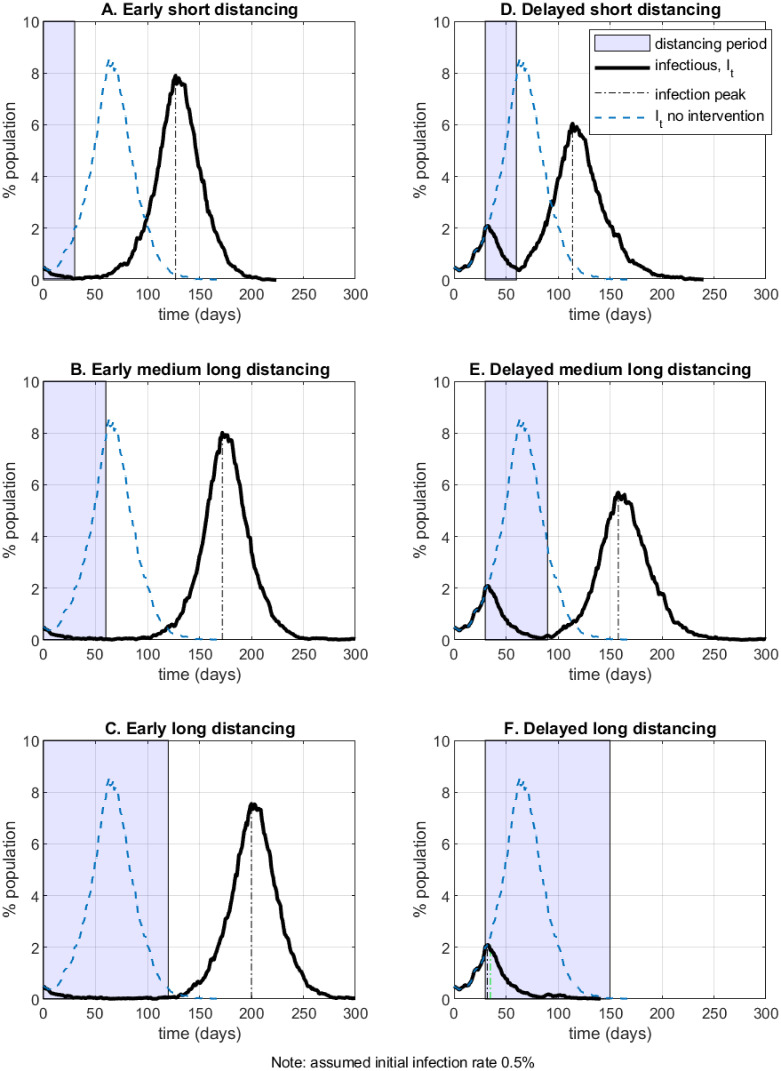
Distancing policy—duration and timing.

Fig 4 (panels A to F) exhibits six different example scenarios which vary the assumed distancing policy duration (‘short’—30 days; ‘medium-long’ – 60 days; ‘long’—120 days) and the policy timing (‘early’, at *t* = 0; or ‘delayed’, at *t* = 30). At the calibrated parameters, distancing policies of short and medium-long duration fail to contain the epidemic in the simulated outcomes. In panel C, even a 4-month long distancing policy imposed at the 0.5% infection rate mark may only delay the epidemic (this happens in 20% of the simulation runs with different random seeds; in the remaining runs policy C contains the epidemic with 1.6% total infection rate and 0.01% death rate). Scenario C also illustrates how the network path dependency in the NSIR setting relative to the SIR model (which node infects when) matters (see Fig E in the S1 Appendix). In example scenario F the epidemic is successfully suppressed by imposing a sufficiently long (120 days) distancing policy with delay.

The simulation results in Fig 4 show that delaying the introduction of a distancing policy may be beneficial in some cases—the left-side panels with the right-side panels. Intuitively, an appropriately-timed delayed policy can create a two-peaked infection curve (as opposed of a single high peak), which is a form of “curve flattening”. However, such delays may possibly overwhelm a country’s health system capacity (not modeled here) or result in larger economic costs, an issue explored further in Sections 5 and 6.

**Fig 5:**
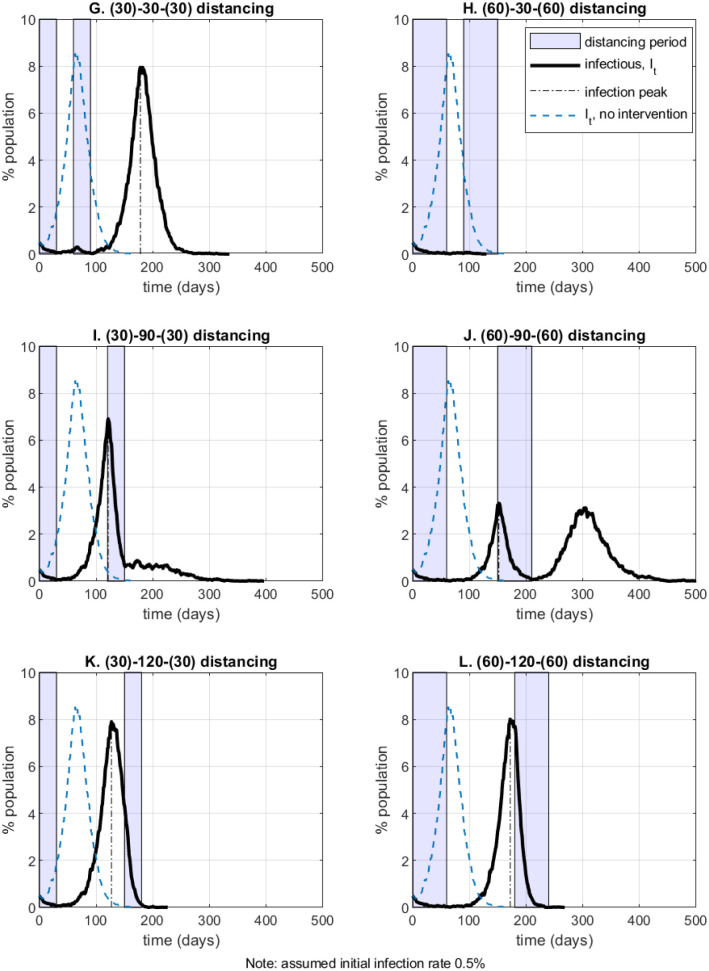
Intermittent distancing policies.

In Fig 5 (panels G through L) I evaluate *intermittent* distancing policies, that is, policies consisting of two separate periods of physical distancing (contacts on social network *Q*), with “back to normal” (contacts on social network *G*) time in between. The notation (*x*)-*y*-(*x*) in the panel captions means *x* days of distancing, followed by *y* days of policy relaxation, followed by *x* days of distancing again. Current events as of May 2020 suggest that such intermittent policies may be easier to implement or enforce politically in many countries.

There are two main takeaways from the hypothetical policy evaluations in Fig 5 (see also Table 3 in Section 6). First, two shorter distancing periods spaced farther apart (as in panels I or J of Fig 5) could be more effective in flattening the infection peak compared to a single longer distancing period imposed early on (panels B and C of Fig 4) or compared to two early distancing periods close to each other (panel G of Fig 5). On average, scenario I results in 7% (6.6%) less total infections and 4% (2.5%) lower annualized economic cost than scenario B (scenario G). Second, the policy timing matters a lot—for example, longer distancing period early on, or a second period of distancing that is too late, are less effective in flattening the infection peak (compare panels K and L with panels H and J in Fig 5).

#### 4.2.3 Lockdown policies

**Fig 6:**
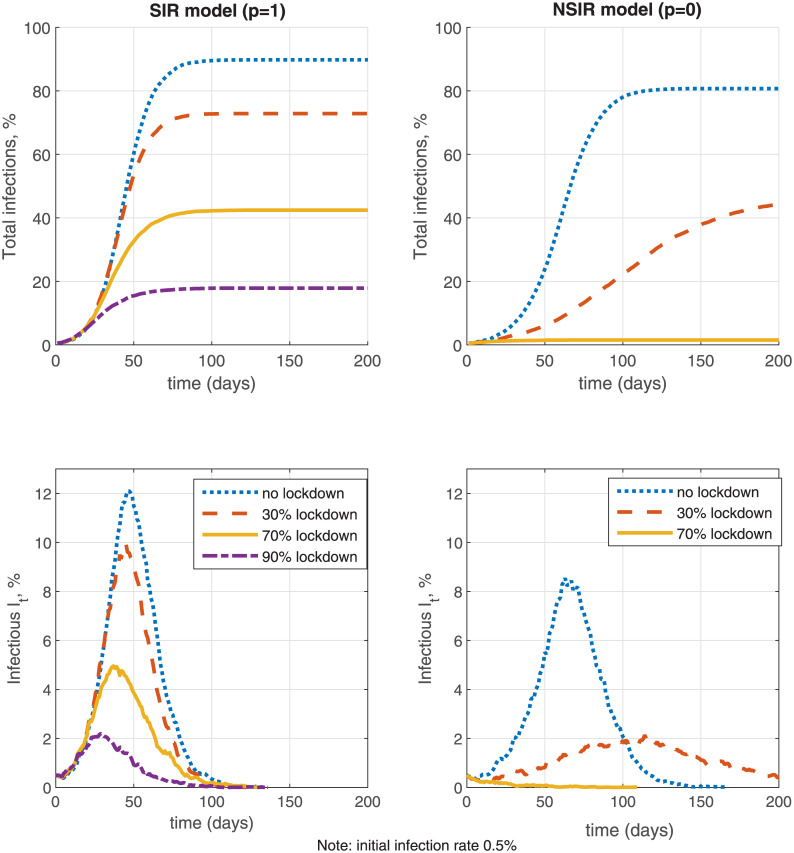
Lockdown effectiveness.

On Fig 6 I simulate and compare the effectiveness of a lockdown policy with fraction of locked down agents *λ* equal to zero (no lockdown), 30%, 70% and 90% for the pure SIR model (*p* = 1) and the network-only NSIR model (*p* = 0). The lockdown intervention is defined as in Section 3 and is assumed indefinitely long (there is no testing or contact tracing). The simulations are initialized with 0.5% initial infection rate. The main difference between the lockdown and the distancing policies explored in the previous sub-section is that lockdowns affect both the population-level transmission and the network-level transmission, by reducing the contact rate for *fraction* *λ* of the population, see (11). In contrast, the distancing policies defined in Section 4.2.2 only affect network-level transmission (replacing the graph *G* by *Q*) but apply to *all* agents.

In the SIR model with only population-level uniform mixing (*p* = 1, the left-side panels of Fig 6), the effectiveness of lockdowns is limited since both the numerator and denominator in the infection probability term $\beta \frac{\left(1-\lambda \right)I_{t}}{\left(1-\lambda \right)N-F_{t}}$ in (11) are reduced nearly proportionately for low death counts *F*_*t*_ and hence the reproduction number among the agents not in lockdown remains high. Expression (11) assumes that the individual contact rate for agents not in lockdown remains the same as without lockdown; the lockdown effectiveness would be higher if the contact rate is also reduced, e.g. as in [3]. The simulation shows that even a 90% (indefinitely long) lockdown only reduces the infection rate and peak but does not eliminate the epidemic. In contrast, in the NSIR model with network-level transmission only (*p* = 0, the right-side panels of Fig 6), a mild *λ* = 30% lockdown flattens the infection curve significantly by taking out many potential contacts and vectors of transmission while a moderate 70% (indefinitely long) lockdown contains the epidemic. While these are simulated examples, the robust implication is that the global vs. network-level mixing degree (the parameter *p*) plays a key role in lockdown efficiency.

**Fig 7:**
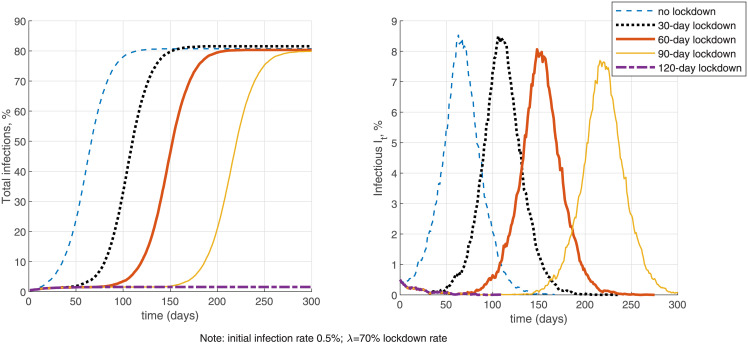
Lockdown length.

In Fig 7 I further investigate the effectiveness of a 70% lockdown with different finite durations in the network-only NSIR model, *p* = 0, staring from a 0.5% initial infection rate. Without testing (the left-side panels), lockdowns with duration shorter than 120 days mostly delay the infection peak but do not contain the epidemic. Summary Table 3 in Section 6 further quantifies that a 30-day lockdown only reduces total infections by 0.5% and the infection peak by 5% on average. A longer 90-day lockdown in contrast reduces total infections by 49%, the infection peak by 52% and total deaths by 48% on average, relative to the no-intervention benchmark. These averages are, however, composed by two types of outcomes—the 90-day lockdown either fully contains the epidemic or only delays the peak and makes a small dent in infections and deaths (see Fig 7 for the latter case). These results do depend on the assumed initial infection rate (0.5% in Fig 7)—the minimum required lockdown period is shorter if started at a lower infection rate.

**Fig 8:**
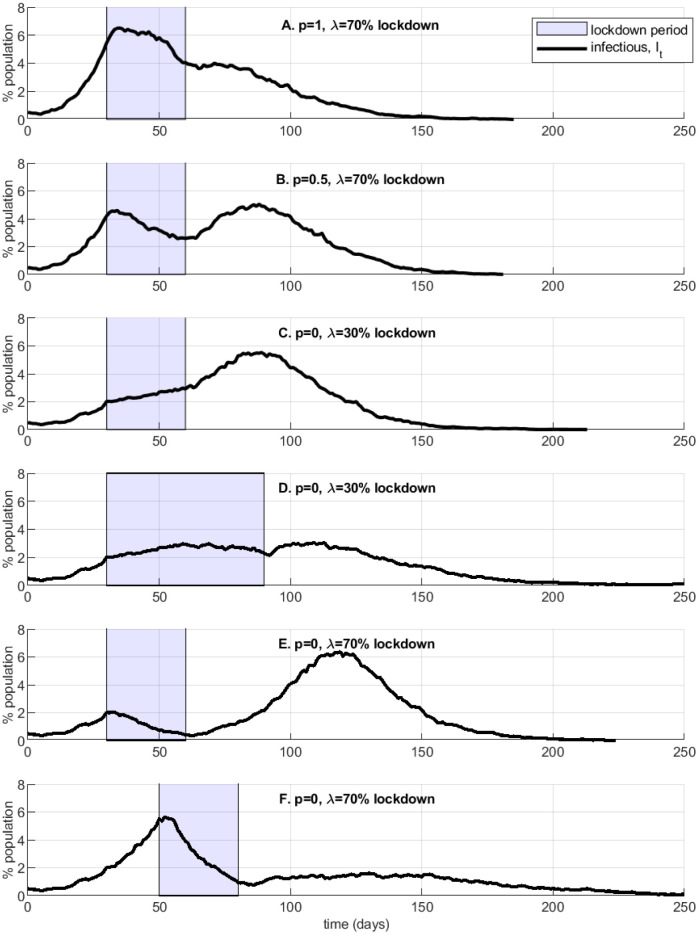
Lockdown success or failure.

Next, Fig 8 explores several illustrative example simulations of lockdowns followed by relaxation. A short or inefficiently timed (panels A, B, C, E) and/or lax (C, D) lockdown may result in (i) a slow and prolonged decline in infections, with asymmetry in the growth rates of infection ramp-up vs. decrease (e.g., as observed in Italy or Spain) and (ii) a second, larger and/or longer epidemic wave (panels B, E). In contrast, in panel F a strict well-timed lockdown reduces the infection rate significantly below the peak, although in this simulation the epidemic carries on at lower intensity for a long time.

#### 4.2.4 Lockdown exit—role of testing and contact tracing

**Fig 9:**
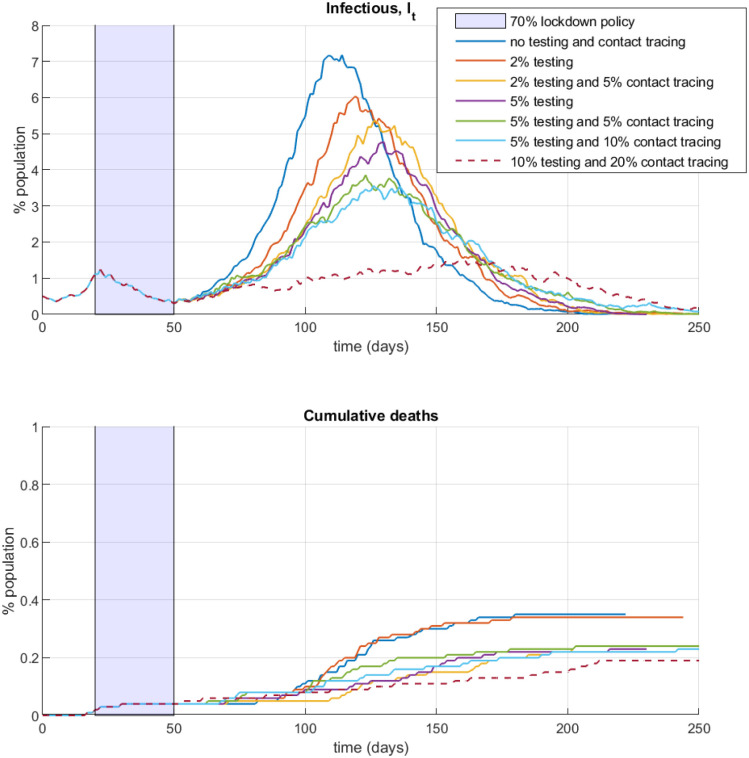
Lockdown exit, testing and contact tracing.

I next perform simulations to investigate the complementarity between lockdown policies and follow-up testing and contact tracing. Specifically, Fig 9 considers a 30-day lockdown for 70% of the population in the NSIR model with *p* = 0. I simulate alternative lockdown exit scenarios, varying the testing and/or contact tracing rate. All agents who test positive are assumed to be quarantined or (self-)isolating and interact on the reduced degree close-contacts graph *Q* with average node degree 1 defined in Section 4.1.

The values for the testing rate *θ* and the contact tracing rate *ϕ* used in the simulations on Fig 9 are hypotheticals, corresponding to continuous mass testing and contact tracing. The results show that opening up social and economic interactions after a relatively short lockdown without testing or with little testing in place can soon result in a new, higher infection peak and larger total number of deaths, because of the large remaining fraction of susceptible persons. Second, testing and contact tracing are complementary—mass testing combined with intensive contact tracing can significantly mitigate the epidemic while mass testing alone may be insufficient to prevent a new infection wave. The simulations suggest that, for the calibrated parameters, very high rates of testing and tracing are needed to prevent a new peak after a short 30-day lockdown. Prolonging the lockdown period (assuming that moving it forward in time is not possible) is likely to be more effective in reducing infections and deaths although it carries larger economic (and possibly political) costs.

In the Appendix (Fig D in S1 Appendix) I perform the same set of simulations for the SIR model with global transmission only, *p* = 1. Comparing Fig 9 with Fig D in S1 Appendix reveals that testing and contact tracing are less effective with population-level mixing compared to in the network-contacts model, for the reasons discussed in Section 4.2.1.

### 4.3 Behavioral responses

**Fig 10:**
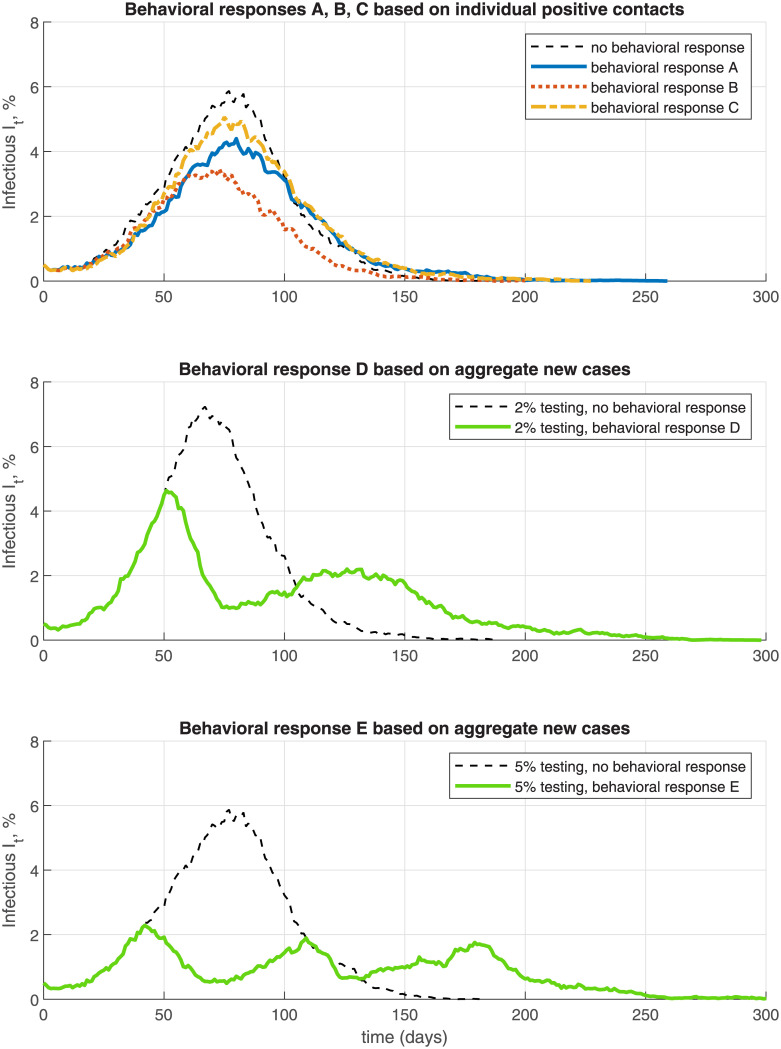
Behavioral responses—endogenous distancing and relaxation.

The NSIR model allows incorporating behavioral responses by the agents, based either on individual-level information (from their own social contacts) or aggregate-level information. In Fig 10 and Table 3 I analyze five simulated scenarios of behavioral responses in the network-only NSIR model, *p* = 0. Behavioral response scenarios A, B and C assume testing rate *θ* = 0.05 and model a (e.g., fear-driven) reduction in an agent’s number of contacts if the agent learns that one of his social contacts has tested positive, that is, if *x*_*jt*_ = *P* for some agent *j* ∈ *C*_*G*_(*i*). This behavioral response works as self-triggered contact tracing.

Formally, a susceptible agent *i* for whom ∃*j* ∈ *C*_*G*_(*i*) with *x*_*jt*_ = *P* at some time *t*, switches to a lower-degree social network $\tilde{Q}$ with contacts $C_{\tilde{Q}}\left(i\right)$ where $\tilde{Q}$ is a sub-network of *G* with lower degree per each node. The median degree of network $\tilde{Q}$ is set to 5 in simulation A and to 1 in simulation B. Scenarios A and B assume a permanent switch, to assess the upper bound of the effect. Compared to the baseline setting, Fig 10 (a single simulation path) and Table 3 (average over 100 simulation paths, see Section 6.1) show that these behavioral responses reduce the infection rate, peak and death toll by significant amounts. The total number of infected is reduced on average by 25% in scenario A and 48% in scenario B; the infection peak is reduced by 45% and 60% respectively, and the total death count is reduced by 17% and 41% respectively (see Table 3).

Behavioral response simulation C (see Fig 10 and Table 3) assumes that the switch to the restricted-contacts network $\tilde{Q}$ is temporary. Specifically, a susceptible individual *i* with *x*_*it*_ = *S* switches to graph $\tilde{Q}$ only for the times *t* for which s/he has a social contact who has tested positive and still in state *P*, i.e., ∃*j* ∈ *C*_*G*_(*i*) with *x*_*jt*_ = *P* while *i* uses the baseline social network *G* otherwise. Such adaptive behavior still lowers the infection peak but the reduction in the overall infection and death rates is smaller compared to that in simulations A and B—15% reduction in total infections and 10% reduction in total deaths (see Table 3).

In behavioral response scenarios D and E (Fig 10, middle and bottom panel and Table 3) I assume that agents react to new positive cases in the population. Specifically, the susceptible agents observe the infection aggregates and choose to reduce their contacts by switching from graph *G* to graph $\tilde{Q}$ if there is a large increase *Δ* in new active cases *P*_*t*_ (*Δ* is set to 100 per 10,000), over the preceding 20 days. The agents revert back to contact graph *G* if there are less new cases than the threshold *Δ* over the preceding 20 days. Simulation D uses a 2% testing rate *θ* while simulation E uses 5% testing rate. Fig 10 shows that these behavioral responses result in multiple but low infection peaks, corresponding to alternating periods of endogenous distancing and relaxation. Compared to the scenarios with 2% or 5% testing only and no endogenous behavioral response, the behavioral response scenarios D and E reduce total infections by on average 16% and 25%, the infection peak by 34% and 63%, and total deaths by 7% and 22%, respectively (see Table 3).

### 4.4 Superspreaders

As explained earlier, the effective reproduction number in the NSIR model depends on the network structure and the social contacts of the currently infectious nodes. To illustrate this point further I explicitly look at the role of superspreaders, that is, nodes with a large number of edges. Specifically, I take the baseline no-intervention *p* = 0 simulation from Fig 3 and compute the percent increase in the effective reproduction number $R_{t}^{NSIR}$, as defined in (7), registered immediately after a ‘superspreader’ node becomes infectious (see row 3 in Table 2).

**Table 2:** Impact of superspreaders on the reproduction number $R_{t}$.

superspreader node #	34	29	18	19	36	57	22
node degree	200	188	180	142	138	131	124
$R_{t}$ change from superspreader	9.9%	3.0%	2.2%	2.6%	2.1%	5.1%	5.6%
average preceding $R_{t}$ change	.006%	−.01%	.008%	−.04%	.003%	−.04%	−.05%

I define as superspreaders the ten nodes with largest degree in the baseline graph *G*. From these ten nodes, seven become infected in the simulation, listed in Table 2. The immediate change (increase) in $R_{t}$ because of a superspreader node turning infectious (row 3 in Table 2) is compared to the average preceding $R_{t}$ change (computed as the average change in $R_{t}$ over the preceding 10 model time-steps / state transitions) in row 4. These results show that superspreaders can lead to significant ‘jumps’ in $R_{t}$ in the NSIR model. In contrast, in the SIR model $R_{t}$ changes continuously no matter which node becomes infections since only the total number of currently infectious nodes *I*_*t*_ matters for the effective reproduction number, see (5).

Fig A in the S1 Appendix illustrates further the importance of superspreaders and node degree heterogeneity in the NSIR model, compared to the SIR model with population-level transmission. Fig A in S1 Appendix compares the infection curves resulting from a single initial infectious person who is either a superspreader (node 34 with degree 200) or an average spreader (node 21 with degree 10). I do this for *p* = 1 (random matching, SIR setting), *p* = 0.5 (mixed NSIR) and *p* = 0 (network-only NSIR). In the SIR setting the identity of the initial spreader (or any later one) has no effect on the infection dynamics by construction—only the total number of infectious *I*_*t*_ matters. In contrast, in settings with network-transmission (*p* < 1) an early superspreader results in much earlier and higher infection peak. In the *p* = 0 setting the initial superspreader node generates large number of secondary cases very quickly, who in turn infect others, leading to 758 infected nodes (7.6% of the population) at *t* = 60 as opposed to only 24 infected nodes at *t* = 60 in the simulation with average initial spreader. These examples highlight the necessity for quickly identifying superspreaders or for restricting the situations in which superspreader scenarios are common (e.g., mass gatherings, bars, cruise ships, etc.), as also documented in the medical literature (e.g., [13] or [14]).

### 4.5 Effective reproduction number

I compute and compare the effective reproduction number $R_{t}$ across different model scenarios. In general, for any scenario, with corresponding endogenous *S*_*t*_, *I*_*t*_ and *E*_*t*_, we can compute (agents who tested positive, if any, are included in *I*_*t*_):

$$\begin{array}{c} R_{t}^{NSIR}=\frac{\sum_{i\;\text{s.t.}\;x_{it}=S}\text{Prob}\left(x_{it^{\prime }}=E|x_{it}=S\right)}{rI_{t}}\text{.} \end{array}$$

**Fig 11:**
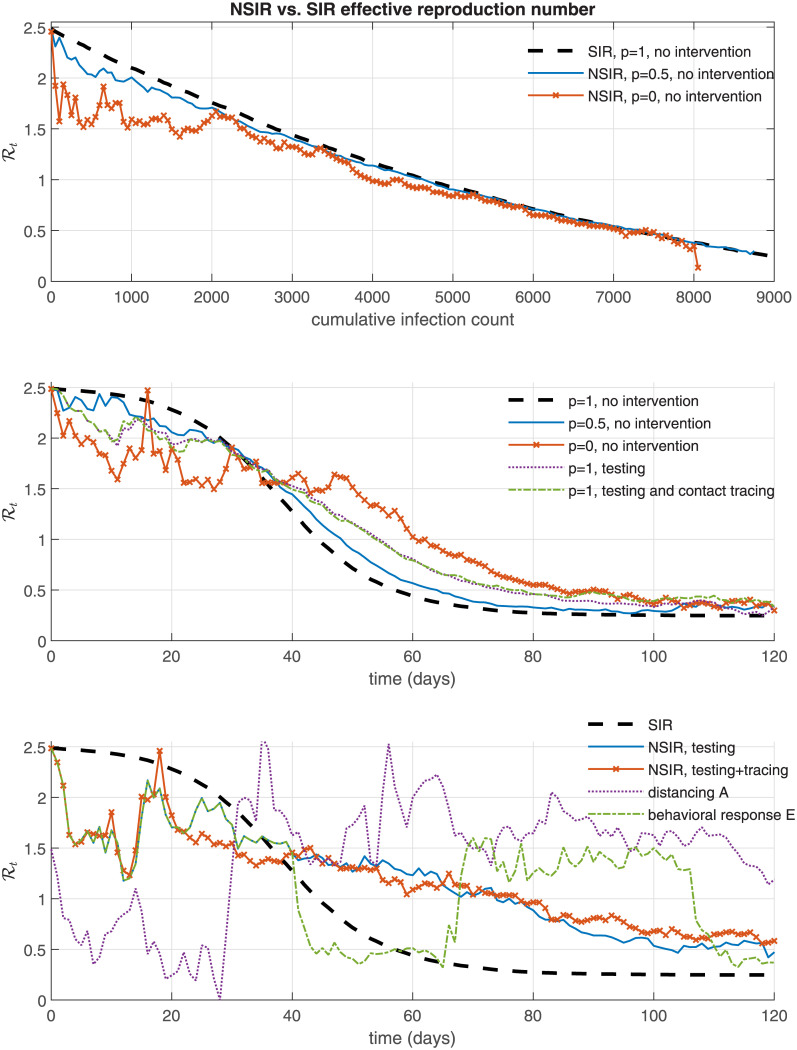
NSIR model—effective reproduction number $R_{t}$.

The top panel of Fig 11 plots the reproduction number of the SIR model (*p* = 1) vs. the NSIR model with network transmission only (*p* = 0) in the absence of any interventions or behavioral responses. The lines in the top panel are plotted against cumulative infection count, as analyzed in Examples 1-3 above. The infection count is not equally spaced in calendar time since there are many new infections when the disease is peaking than in the its early or late stages. The figure shows that for the chosen modified Barabasi-Albert graph *G* the NSIR model has lower reproduction rate $R_{t}$ than the SIR model, with the gap being the largest in the early stages (see Appendix B for more formal discussion on comparing the reproduction numbers in the SIR vs. NSIR models).

The middle and bottom panels in Fig 11 plot the reproduction number $R_{t}^{NSIR}$ over actual calendar time (days) for several of the simulation scenarios considered in the previous sections. Compared to the SIR model (*p* = 1) baseline (the thick dashed line on Fig 11), network-level transmission (*p* < 1) ‘flattens’ the reproduction number—for the assumed social network *G*, the value of $R_{t}^{NSIR}$ is initially below that of the SIR model but it is higher later on (after approximately 60 days on the figure) and may stay around 1 for a prolonged time if the infection rate is slowed down by testing and contact tracing (see the bottom panel). The impact of distancing policies in bringing $R_{t}^{NSIR}$ below 1 is fast and strong, however, the Figure also shows that, when the policy is lifted, the reproduction number may quickly rise above 1 again.

## 5 Economic module

As a simple illustration of the economic costs analysis of the COVID-19 epidemic using the NSIR model, I follow [11] to define and compute an index of economic activity based on the number and relative productivity of active vs. quarantined or sick agents in the economy. Clearly this measure is very rough and excludes indirect (e.g., additional costs from deaths and hospitalization or psychic costs), long-term (job loss, inability to pay debts, destruction of employment attachment), sectoral (e.g., hospitality vs. IT), or general equilibrium effects associated with the (duration of) epidemic or lockdown policies.

**Fig 12:**
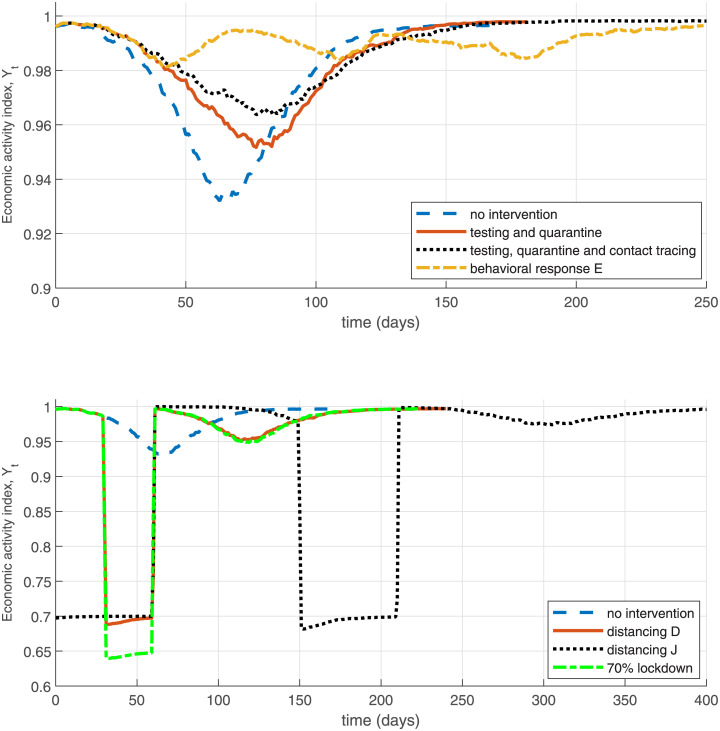
Economic impact illustration—NSIR model.

Define the following simple index of economic activity over time, *Y*_*t*_ which keeps track and varies with the numbers of active and healthy agents vs. locked-down / quarantined agents vs. sick or dead agents.

$$\begin{array}{c} Y_{t}=\frac{1}{N}\left[\left(1-\lambda \right)\left(S_{t}+E_{t}+R_{t}+\alpha I_{t}\right)+\lambda \rho \left(S_{t}+E_{t}+R_{t}+\alpha I_{t}\right)+\rho \alpha P_{t}\right] \end{array}$$

where *λ* ∈ [0 1] is the fraction of agents in lockdown, *ρ* ∈ (0, 1) is the factor with which the productivity of locked down agents is reduced, and *α* is the fraction of infectious agents (*I*_*t*_ or *P*_*t*_) who are asymptomatic (assumed as productive as healthy agents). The rest, 1 − *α* of sick agents are assumed to have zero productivity. All “tested positive” agents are assumed in quarantine (productivity *ρ*). In the simulation results reported in Table 3 and Fig 12 I assume *ρ* = 0.5 as in [11] and *α* = 0.18 as estimated in [30]. The lockdown rate *λ* is a policy variable. A value of 1 for the index *Y*_*t*_ is interpreted as “normal times”, that is, all agents being healthy and fully productive.

Fig 12, the top panel plots the economic index for the NSIR model with network transmission (*p* = 0) and the scenarios in Fig 3 in Section 4.2.1. In the middle and bottom panel I illustrate the effect on *Y*_*t*_ of various policies defined earlier. In these simulations it is assumed that during the distancing period all agents’ productivities are reduced by the factor *ρ*_*D*_ = 0.7. These results are just illustrative and assume large economic costs from broad lockdown or distancing (self-isolation).

Table 3 in Section 6 reports the economic loss measured by the index *Y*_*t*_ across the multiple scenarios considered. Specifically, column “GDP loss” reports the average losses, compared to the baseline *Y*_*t*_ = 1 and annualized to account for the different durations of the epidemic. Column “max GDP fall” in Table 3 displays the largest decrease in the economic index *Y*_*t*_ over the epidemic duration. As expected, because of the mandated reduction in production, the distancing or lockdown scenarios entail the largest average (up to 9% annualized decrease in *Y*_*t*_) and maximum economic losses (up to 35% decrease), with the losses increasing in the intervention duration. The second largest economic costs are observed in the no-intervention scenarios (7–9% maximum decrease in *Y*_*t*_), because they result in a large share of infected agents who are assumed less productive. In contrast, the lowest economic losses result in the testing / contact tracing and behavioral response simulation scenarios, where the combination of no mandated lockdown and low infection shares mitigates economic costs.

Clearly, these results should be interpreted only as illustrative of the productivity losses and the trade-off of between lockdown/distancing vs. infections/deaths since only direct reductions in productivity or output are considered and multiple other factors are omitted: additional cost of deaths, healthcare costs, job losses or (except in the behavioral response scenarios) reductions in economic activity due to fear (e.g., restaurants, travel), etc.

## 6 Summary and robustness

### 6.1 Summary of results

Table 3 summarizes the simulation results from the previous sections (see there for the corresponding discussion). Each Table row reports averages over 100 simulations with the same model parameters and policy setting but different pseudo-random number generator seeds that are held constant across the rows (scenarios).

**Table 3:** Summary of simulation results.

Scenario	total infected	total deaths	peak infectious	peak day^1^	epidemic duration^1^	GDP loss^2^	max GDP fall^3^
no intervention, *p* = 1	89.5%	0.34%	11.7%	47	145	1.3%	9.3%
no intervention, *p* = .5	87.7%	0.33%	10.8%	49	150	1.3%	8.6%
no intervention, *p* = 0	81.6%	0.31%	8.5%	58	170	1.2%	6.7%
all simulations below use the NSIR model with *p* = 0							
mass testing, *θ* = 2%	77.4%	0.28%	7.4%	62	182	1.2%	6.0%
mass testing, *θ* = 5%	70.6%	0.26%	5.8%	69	206	1.1%	4.8%
5% testing and 10% contact tracing	64.6%	0.24%	4.9%	70	221	1.1%	4.0%
accelerating testing^4^	77.6%	0.29%	7.6%	60	173	1.2%	6.1%
distancing policy A	78.6%	0.30%	7.9%	110	219	3.7%	30.3%
distancing policy D	75.1%	0.28%	4.9%	99	254	3.6%	31.7%
distancing policy E	73.0%	0.28%	4.3%	131	330	6.1%	31.7%
distancing policy F	24.5%	0.09%	3.2%	48	151	8.6%	31.7%
distancing policy I	68.8%	0.26%	7.5%	111	243	5.9%	33.2%
distancing policy J	31.2%	0.12%	3.6%	79	173	7.6%	31.6%
distancing policy L	36.3%	0.14%	4.0%	76	152	7.4%	31.1%
lockdown, 70%, 30 days	81.1%	0.31%	8.1%	101	214	4.1%	35.3%
lockdown, 70%, 90 days^5^	30.4%	0.12%	3.1%	68	163	8.6%	35.3%
lockdown, 70%, 90 days, 5% testing	15.2%	0.06%	1.4%	45	132	7.7%	35.3%
behavioral response A^6^	61.6%	0.23%	4.9%	66	207	1.0%	4.0%
behavioral response B^6^	43.5%	0.17%	3.6%	58	193	0.8%	3.0%
behavioral response C^7^	68.8%	0.25%	5.5%	65	218	1.1%	4.5%
behavioral response D^8^	64.8%	0.24%	4.9%	45	292	1.1%	3.9%
behavioral response E^8^	52.6%	0.19%	2.3%	46	423	1.0%	1.9%

Notes: All values are averages over 100 simulations with different random seeds. The same random seeds are used in each scenario. Each simulation is initialized at 0.5% infection rate (*I*_0_ = 50). 1. The infection peak and duration are computed relative to that initialization. 2. GDP loss = annualized decrease in the economic index *Y*_*t*_ defined in Section 5.1; 3. max GDP fall = largest instantaneous fall in *Y*_*t*_; 4. Initial rate *θ* = .01 increasing by 10% every 10 days; 5. The epidemic is contained (approx. 2% total infected) in 64% of these simulations and in 80% of the 90-day lockdown, 5% testing simulations in the next row. 6. agents with contacts who tested positive reduce their graph degree (#contacts) on average in half (A) or 10 times (B) thereafter; 7. agents reduce their #contacts only during periods in which they have a contact who tested positive; 8. agents reduce their graph degree upon observing large number of new active cases over the previous 20 days.

### 6.2 Alternative specifications and robustness

There is still a lot of uncertainty and variation in the COVID-19 epidemiological parameter estimates and other model ingredients in the current early state of the literature. The baseline parameters I have used in this paper are believed to be current as of early May 2020, however, depending on different data sources and clinical studies, different authors use different values for the removal and mortality rates tied in the model to the parameters *r* and *μ* (see Section 2), e.g., larger mortality rate or slower removal rate. Note that the observed removal rate may be ‘contaminated’ by policy effects (e.g., if health authorities isolate symptomatic individuals) so using data from policy-treated time periods and locations to estimate the epidemiological parameters should be treated with caution.

**Fig 13:**
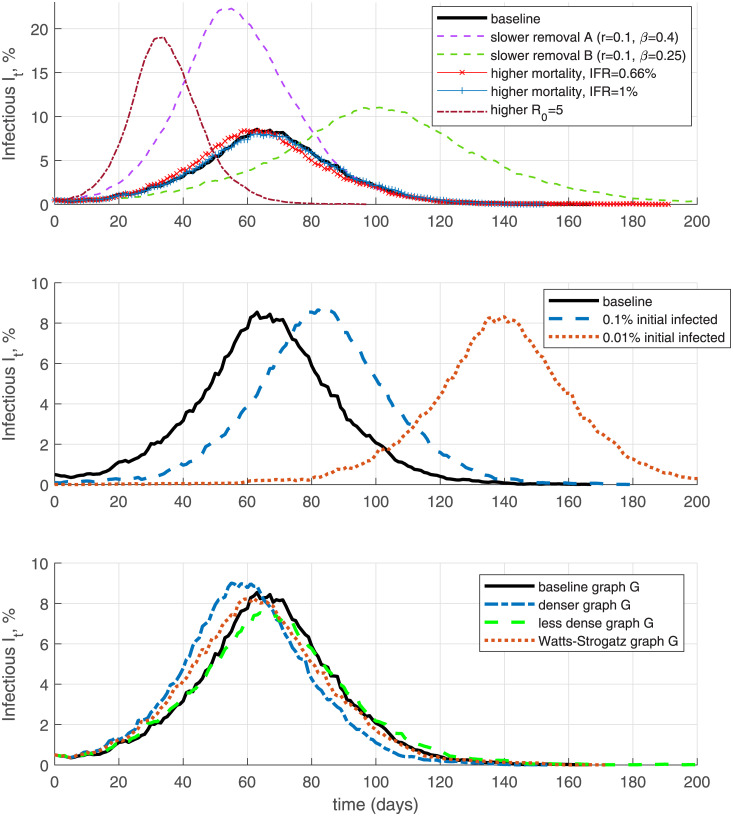
Alternative specifications.

In Fig 13 (top panel) I explore the implications of using alternative epidemiological parameters relative to the baseline calibration in Section 4.1. In simulation ‘slower removal A’ I keep the initial doubling time the same, so *β* − *r* = 0.3 but assume lower removal rate *r* = 0.1, corresponding to a longer, 10-day on average, infectious period instead of 5 days (this raises the SIR *R*_0_ to 4). This creates a higher infection peak and shifts the active infected curve *I*_*t*_ forward in time, since there is slower exit from state *I*. Alternatively, in specification ‘slower removal B’, I assume *r* = 0.1 but keep *R*_0_ = 2.5 as in the baseline (i.e., use *β* = 0.25). The result is a higher infection peak but the infection rate curve moves back in time as the epidemic spreads slower due to the lower infectiousness rate. A higher mortality rate, *μ* = 0.0066*r* or *μ* = 0.01*r*, corresponding to IFR of 0.66% or 1%, has a very minor effect on the infection curve. It does, however, impact total deaths *F*_*t*max_ (not reported on the Figure) since they are a fraction *μ* of cumulative infections, *F*_*t*max_ ≃ *μ*(*N* − *R*_*t*max_). Finally, I keep *r* = 0.2 as in the baseline but explore raising the SIR *R*_0_ to 5 (i.e., *β* = 1)—this results in a much earlier and higher infection peak.

The middle panel of Fig 13 displays simulations exploring lower initial infection rates *I*_0_ = 0.1% or *I*_0_ = 0.01%. The result of these alternative initial conditions is largely just a time shift in the infection curve, suggesting that in empirical work it is important to carefully calibrate the initial condition to match the infection peak.

Finally, in the bottom panel of Fig 13 I perform simulations with alternative specification of the social contacts graph *G*—by assuming a higher density of contacts (median node degree equal to 13, instead of 10 in the baseline); lower density of contacts (median node degree 8); or a Watts-Strogatz graph *G* with mean degree 12. The specification using a denser graph moves the infection peak slightly forward and upward in time, while the opposite is true for the specification with less dense graph. Otherwise I find that the shape of the infection curve *I*_*t*_ is not very sensitive to these alternative assumptions about the contacts graph *G*. A necessary step for future empirical work is to calibrate the network *G* using actual data, e.g., as in [17].

## 7 Conclusions

I analyze the combination and interaction of a compartmental epidemiological model and a network model of social contacts (an NSIR model). I explore, via calibration and simulations, how network-based transmission and the network structure affect the epidemic dynamics as well as the outcomes and effectiveness of a broad range of policy interventions and behavioral responses, compared to the standard SIR model with population-level uniform mixing.

I find that viral transmission over a network-connected population can proceed slower and reach lower peak compared to transmission via uniform/random mixing. Network-based viral transmission introduces uncertainty and path dependence in the epidemic dynamics, with important role for bridge links and superspreaders. Testing, quarantine and contact tracing tend to be more effective in the network model, as these policy interventions can quickly isolate infectious nodes with a large number of contacts. Similarly, interventions that can break major transmission vectors across local sub-populations, such as restrictions on non-local travel or bans on mass gatherings are also very effective. Other implications of the NSIR model remain in line with those in the standard SIR models. If lifted early, distancing policies mostly shift the infection peak into the future, while intermittent interventions or endogenous behavioral responses can generate a flattened, multi-peaked infection curve but may have costlier economic consequences by prolonging the epidemic duration.

The main advantage of the network approach, compared to standard aggregate SIR-type models is that the NSIR model captures heterogeneity and locality of social contacts as possible vectors of transmissions. This allows a micro-level, agent-based modeling of health and economic policy outcomes and individual behavioral responses. In addition, the social contact heterogeneity induces path-dependence and role for superspreaders or clusters in the epidemic dynamics (see [13] or [14] for empirical evidence). The main challenge to the network approach is that, in addition to the standard epidemiological parameters governing disease incubation, infectiousness, recovery and mortality, the specification and identification of the social contacts graph, initial conditions and node path followed by the epidemic require additional attention in future empirical work. Adding further detail, including agent-level, on the economics side of the model can also yield important insights.

## Supporting information

S1 Appendix (PDF)
