# Supplementary material for: A social network model of COVID-19
Source: PLoS One. 2020 Oct 29;15(10):e0240878. doi: 10.1371/journal.pone.0240878 (PMC7595335; doi:10.1371/journal.pone.0240878)
Supplement: S1 Appendix — (PDF) [file pone.0240878.s001.pdf]

S1 Appendix

Table 1. Testing, network vs. uniform transmission

	NSIR (network), $p = 0$ change relative to no testing				SIR (uniform), $p = 1$ change relative to no testing			
testing rate, $\theta =$	*0.1%*	*2%*	*5%*	*10%*	*0.1%*	*2%*	*5%*	*10%*
total infected %	-0.3%	-5.1%	-14%	-30%	-0.2%	-3.6%	-9.9%	-23%
infection peak %	-0.4%	-13%	-31%	-56%	-0.4%	-10%	-25%	-48%
total deaths %	-3.9%	-8.3%	-16%	-30%	-0.6%	-7.1%	-9.2%	-20%
duration, days	-0.6%	+7.6%	+22%	+41%	-0.6%	+4.1%	+13%	+31%

Table 2. Contact tracing, network vs. uniform transmission.

	NSIR (network), $p = 0$ change relative to no contact tracing				SIR (uniform), $p = 1$ change relative to no contact tracing			
tracing rate, $\phi =$	*0.01*	*0.1*	*0.2*	*0.5*	*0.01*	*0.1*	*0.2*	*0.5*
total infected, %	-1.1%	-8.5%	-15%	-27%	-0.4%	-3.8%	-7.1%	-13%
infection peak, %	-2.9%	-16%	-30%	-51%	-0.7%	-6.5%	-12%	-24%
total deaths, %	-1.6%	-8.1%	-11%	-28%	-1.9%	-5.7%	-8.4%	-15%
duration, days	-2.0%	+7.5%	+21%	+45%	+0.2%	+4.3%	+10%	+23%

Note: all results in this table use testing rate $\theta = 5\%$.

Fig A. Role of superspreaders (single initial case, no intervention). Notes: The dashed line corresponds to infection starting from node 21 ('average' spreader with 10 social contacts); the solid red line corresponds to infection starting from node 34 ('superspreader' with 200 contacts). The lines coincide in the SIR model ($p = 1$).

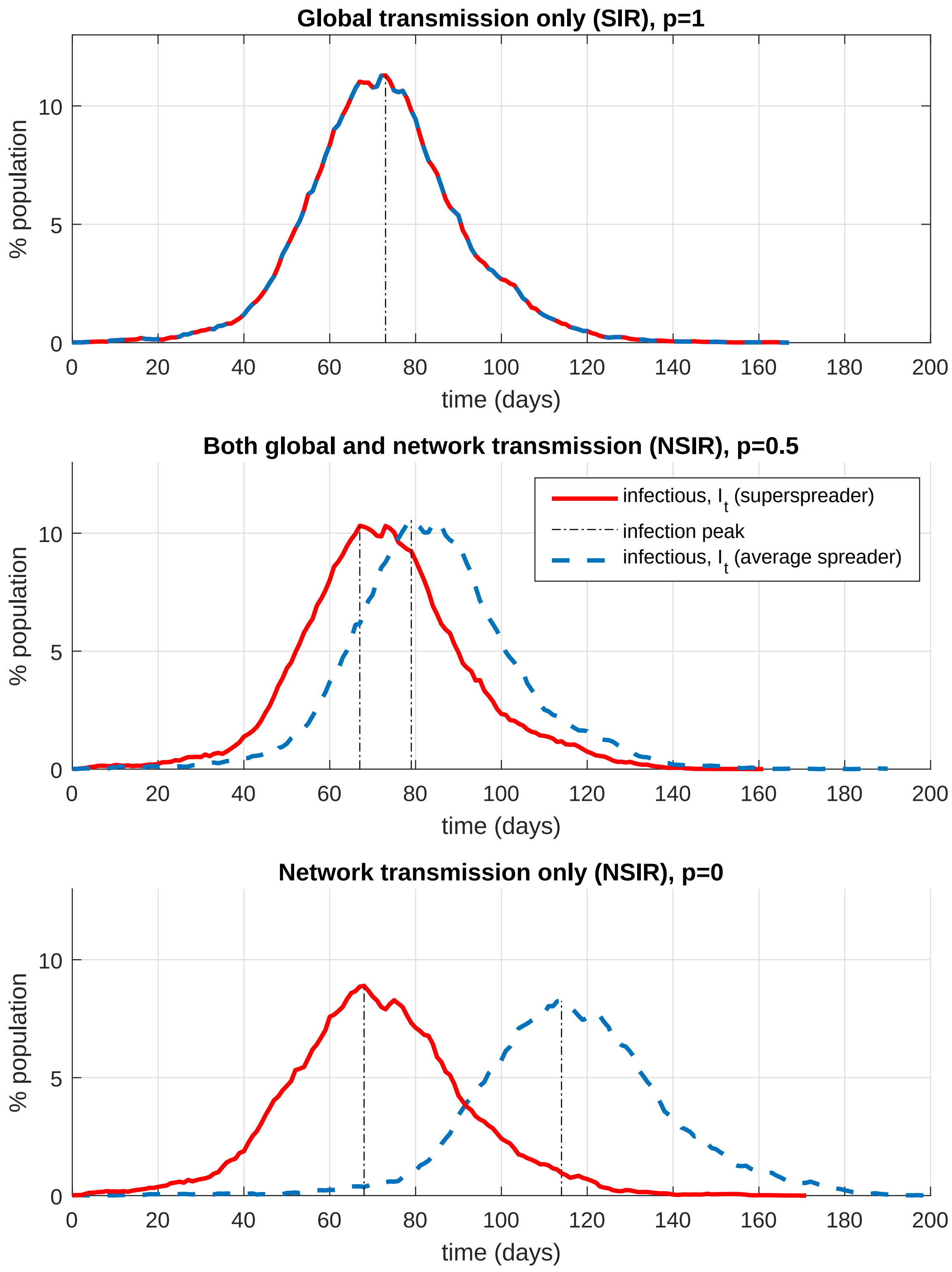

Global transmission only (SIR), p=1
Both global and network transmission (NSIR), p=0.5
Network transmission only (NSIR), p=0
% population
time (days)
0
5
10
0
20
40
60
80
100
120
140
160
180
200
infectious, I_t (superspreader)
infection peak
infectious, I_t (average spreader)

Fig B. Baseline graph G vs. Albert-Barabasi and Watts-Strogatz graphs.

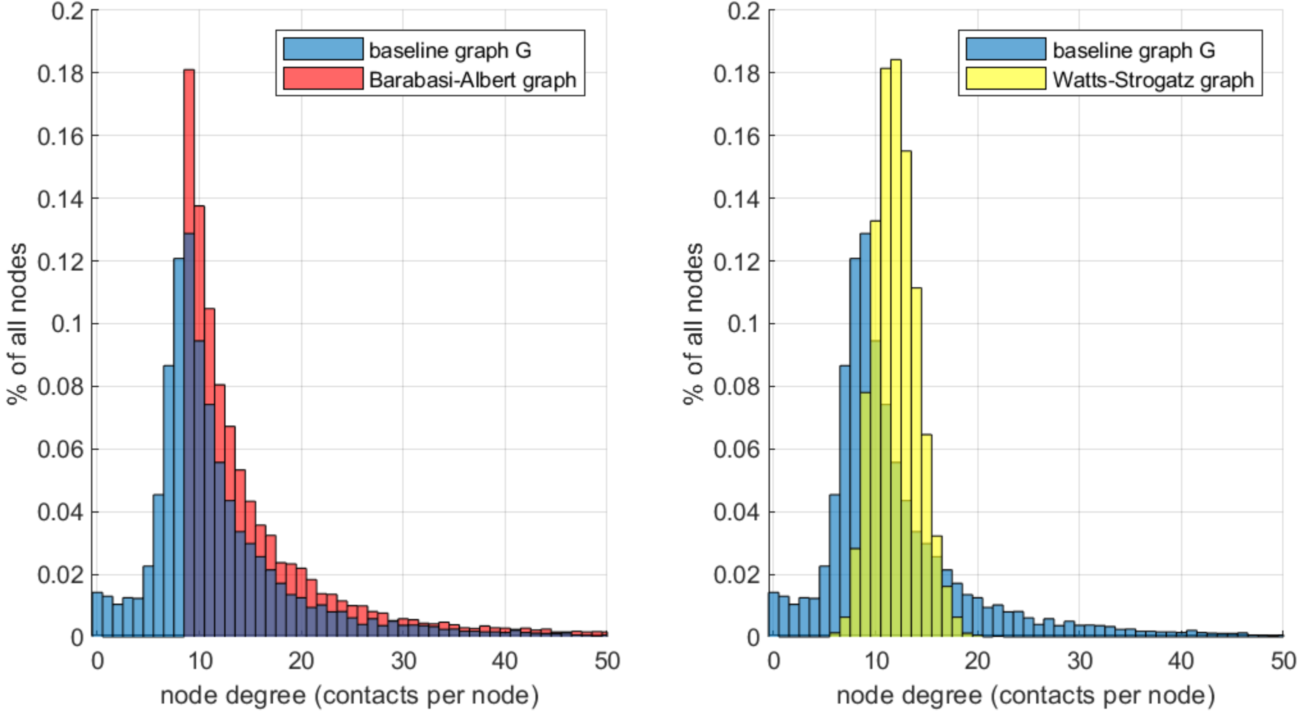
baseline graph G
Barabasi-Albert graph
% of all nodes
node degree (contacts per node)
baseline graph G
Watts-Strogatz graph
% of all nodes
node degree (contacts per node)

Fig C. Baseline graph G and close-contacts graph Q.

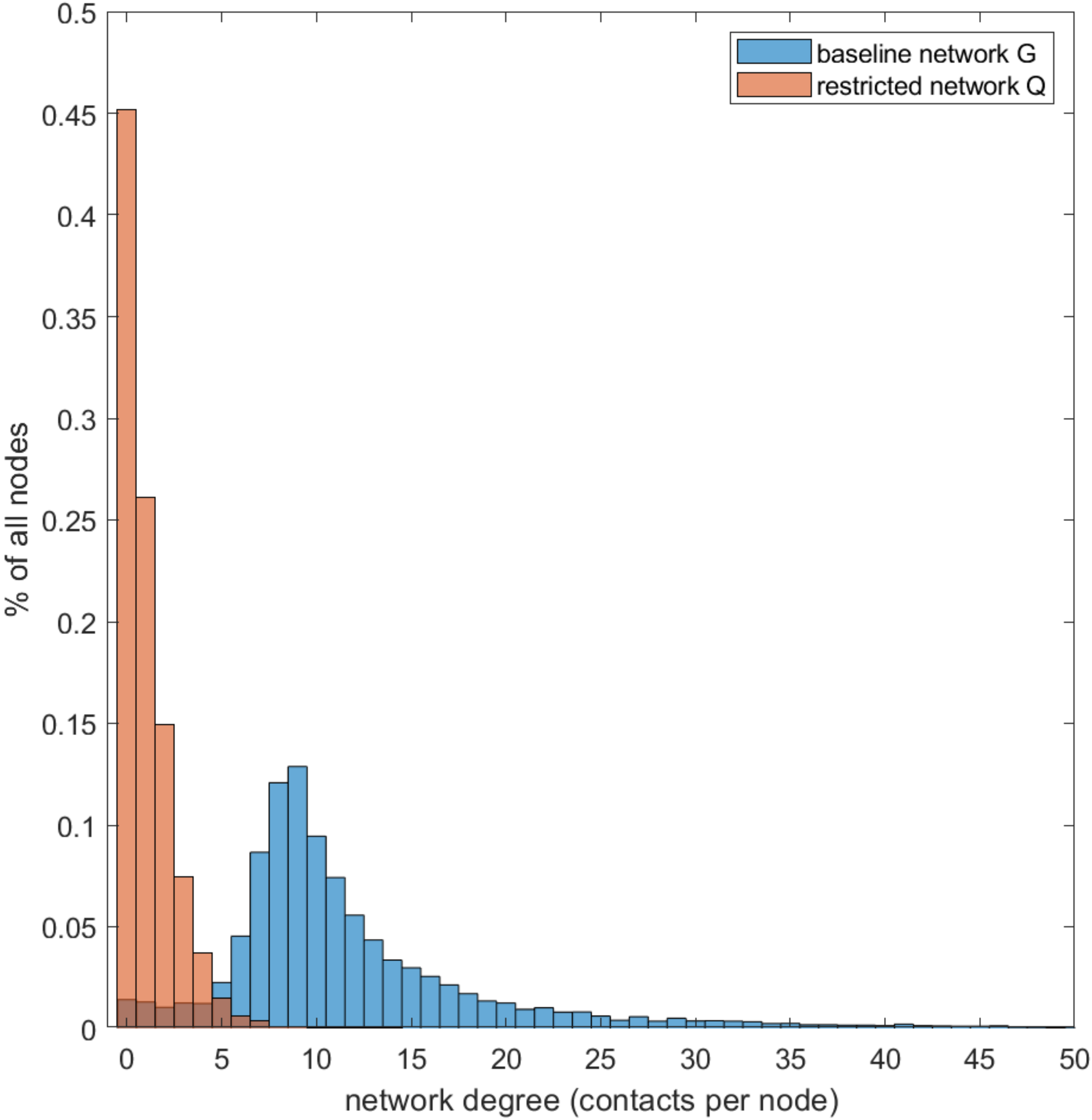
baseline network G
restricted network Q
% of all nodes
network degree (contacts per node)
0
0.05
0.1
0.15
0.2
0.25
0.3
0.35
0.4
0.45
0.5
0
5
10
15
20
25
30
35
40
45
50

Fig D. Lockdown exit, mass testing and contact tracing – global transmission only, $p = 1$.

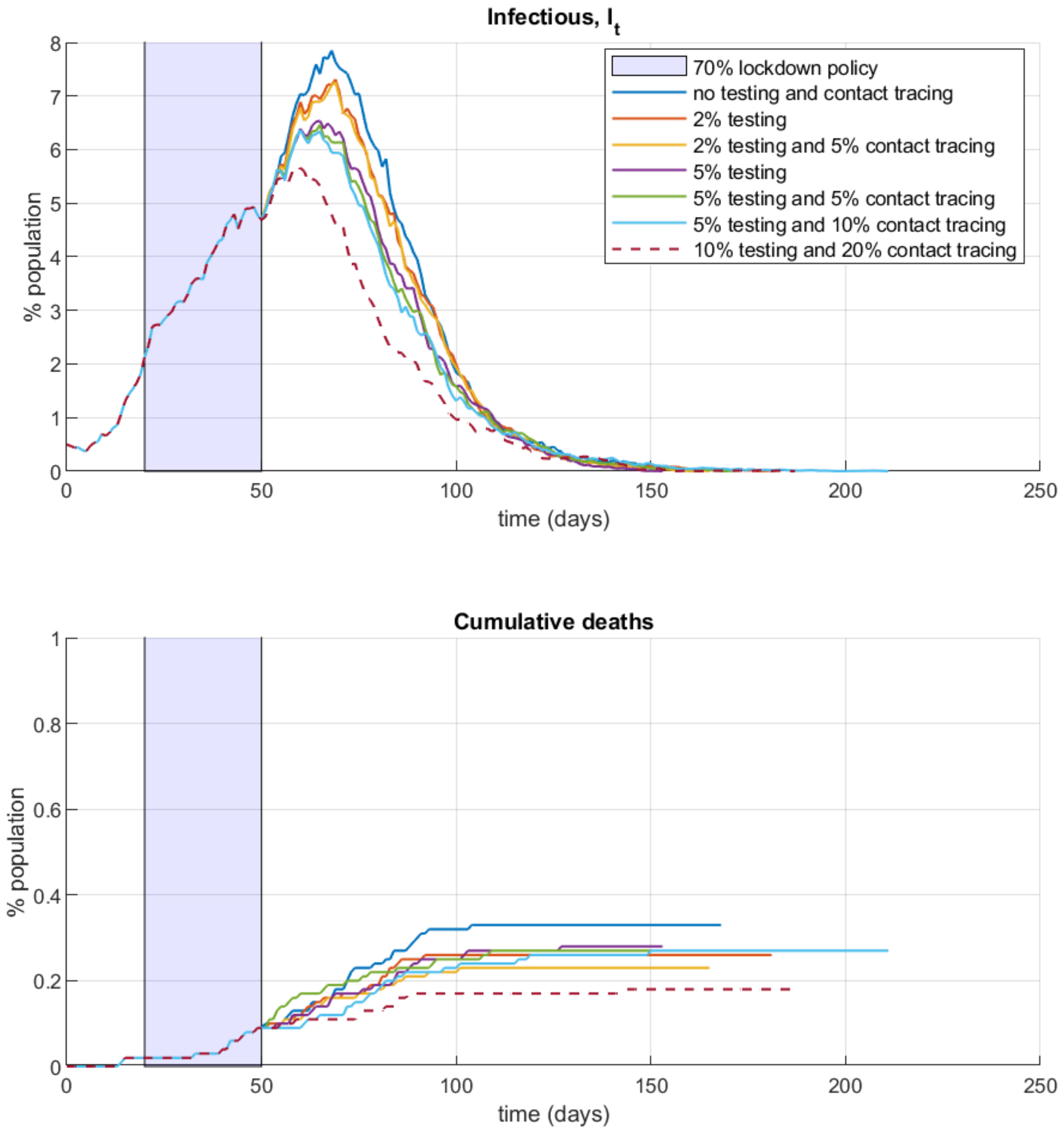
Infectious, I_t
% population
time (days)
70% lockdown policy
no testing and contact tracing
2% testing
2% testing and 5% contact tracing
5% testing
5% testing and 5% contact tracing
5% testing and 10% contact tracing
10% testing and 20% contact tracing
Cumulative deaths
% population
time (days)

Fig E. Network path dependency – 10 different distancing policy J simulation runs.

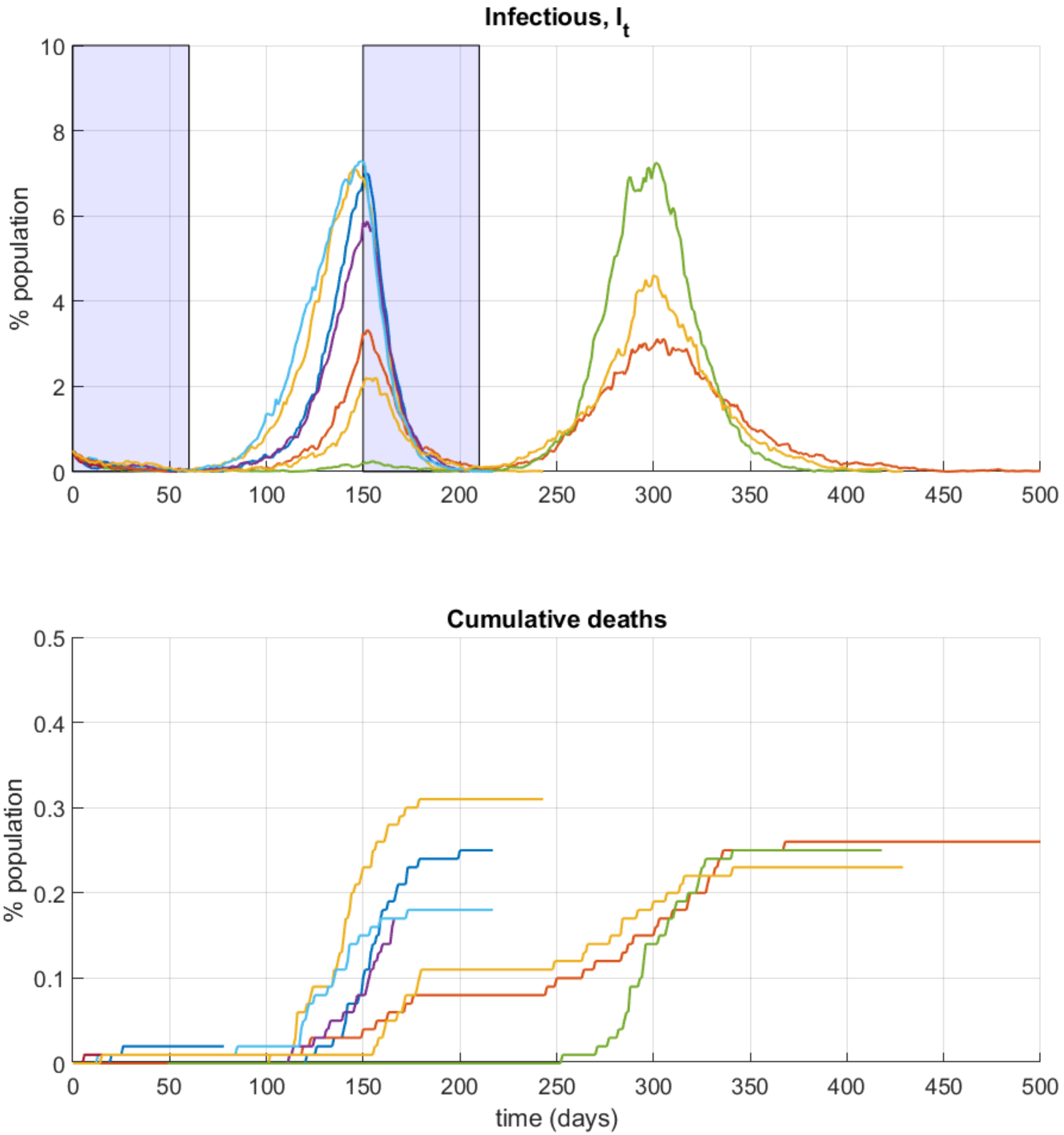
Infectious, I_t
% population
Cumulative deaths
% population
time (days)
